# Transcriptome Analyses Throughout Chili Pepper Fruit Development Reveal Novel Insights into the Domestication Process

**DOI:** 10.3390/plants10030585

**Published:** 2021-03-19

**Authors:** Octavio Martínez, Magda L. Arce-Rodríguez, Fernando Hernández-Godínez, Christian Escoto-Sandoval, Felipe Cervantes-Hernández, Corina Hayano-Kanashiro, José J. Ordaz-Ortiz, M. Humberto Reyes-Valdés, Fernando G. Razo-Mendivil, Ana Garcés-Claver, Neftalí Ochoa-Alejo

**Affiliations:** 1 Centro de Investigación y de Estudios Avanzados del Instituto Politécnico Nacional (Cinvestav), Unidad de Genómica Avanzada (Langebio), Irapuato, Guanajuato 36824, Mexico; fernando.hernandezg@cinvestav.mx (F.H.-G.); christian.escoto@cinvestav.mx (C.E.-S.); felipe.cervantes@cinvestav.mx (F.C.-H.); jose.ordaz.ortiz@cinvestav.mx (J.J.O.-O.); 2 Departamento de Ingeniería Genética, Centro de Investigación y de Estudios Avanzados del Instituto Politécnico Nacional (Cinvestav), Irapuato, Guanajuato 36824, Mexico; lisette02@hotmail.com; 3 Departamento de Investigaciones Científicas y Tecnológicas de la Universidad de Sonora, Universidad de Sonora, Hermosillo, Sonora 83000, Mexico; angela.hayano@unison.mx (C.H.-K.); razoto@gmail.com (F.G.R.-M.); 4 Department of Plant Breeding, Universidad Autónoma Agraria Antonio Narro, Saltillo, Coahuila 25315, Mexico; mathgenome@gmail.com; 5 Unidad de Hortofruticultura, Centro de Investigación y Tecnología Agroalimentaria de Aragón, Instituto Agroalimentario de Aragón-IA2 (CITA-Universidad de Zaragoza), 50059 Zaragoza, Spain; agarces@cita-aragon.es

**Keywords:** capsicum, fruit, gene expression, transcriptome, RNA-Seq, domestication

## Abstract

Chili pepper (*Capsicum* spp.) is an important crop, as well as a model for fruit development studies and domestication. Here, we performed a time-course experiment to estimate standardized gene expression profiles with respect to fruit development for six domesticated and four wild chili pepper ancestors. We sampled the transcriptomes every 10 days from flowering to fruit maturity, and found that the mean standardized expression profiles for domesticated and wild accessions significantly differed. The mean standardized expression was higher and peaked earlier for domesticated vs. wild genotypes, particularly for genes involved in the cell cycle that ultimately control fruit size. We postulate that these gene expression changes are driven by selection pressures during domestication and show a robust network of cell cycle genes with a time shift in expression, which explains some of the differences between domesticated and wild phenotypes.

## 1. Introduction

Chili peppers of the genus *Capsicum* and the Solanaceae family are native to the American continent. Of the approximately 30 chili pepper species, five have been domesticated: *C. annuum* L., *C. frutescens* L., *C. baccatum L.*, *C. chinense* Jacq. and *C. pubescens* Ruiz and Pav [1]. Among these species, *C. annuum* is the most important worldwide as a vegetable and spice crop, and production of this type of chili pepper has been steadily increasing in terms of both area harvested and yield [2]. In addition to their economic importance, chili peppers are a source of antioxidants, such as flavonoids, phenolic acids, carotenoids, and vitamins [3,4], as well as a plant model for studying the genetic and biochemical basis for the synthesis of these compounds [5,6,7]. Capsaicinoids, which are synthesized only in *Capsicum* species, impart pungency to chili peppers and are a focus of active research [8,9,10]. Furthermore, differential expression analysis during chili pepper fruit development allowed the identification of regulatory and biosynthetic genes related to different metabolic processes during fruit development and ripening, including fruit softening, pigmentation, ripening time, and metabolite accumulation [11,12,13].

Chili peppers were domesticated from an ancestral variety, *Capsicum annuum* L. var. *glabriusculum*, locally known as “piquín” or “chiltepín” [14] in northeastern Mexico and/or central-east Mexico [15]. The oldest chili pepper macroremains date to around the time of their first cultivation or domestication in the mid-Holocene, 9000–7000 before present (BP) [15,16]. The exact time of chili pepper domestication is a subject of debate [17], as starch microfossils of domesticated *Capsicum* dating from 6000 BP have been found at seven sites [18], and there is evidence indicating that the fruit size of domesticated genotypes has increased considerably in the last 1500–1000 years BP [17]. The larger fruit size in domesticated compared with wild ancestors is part of the “domestication syndrome” [19].

Domestication, which involves breeding and selection of wild ancestral forms to modify phenotypes for human use, is not only a key achievement of modern civilization [20], but also provides a unique opportunity to identify the genetic basis of adaptation [21]. Examples of studies of plant domestication include maize [22,23,24,25], common bean [26,27], tomato [28,29,30,31], and *Capsicum* [32,33,34,35]. The *Capsicum* genome (≈3.5 Gb) has been sequenced and annotated [36,37]; currently, there are nine genomic assemblies available from the NCBI (https://www.ncbi.nlm.nih.gov/genome/?term=Capsicum accessed on: 17 March 2021), and further sequencing of different genotypes has been reported [38,39]. In particular, the authors of [36] provided insights in order to evaluate the adaptive landscape of cultivated peppers [40] and reported a set of 511 genes that have a strong genomic domestication footprint. On the other hand, using allele-specific expression and network analyses from one domesticated and one wild chili pepper accession, as well as their $F_{1}$ cross, at 40 days after anthesis (DAA), the authors of [41] proposed that gene expression differences associated with the cultivated form could be explained by *cis*-regulatory hubs acting through *trans*-regulatory cascades.

To study the divergence caused by domestication in gene expression profiles during chili pepper fruit development, we examined fruit transcriptomes of six domesticated and four wild accessions using RNA sequencing (RNA-Seq) every 10 days from anthesis until fruiting at 60 days after anthesis (DAA). Our data show that there are significant differences in the mean expression profiles of domesticated and wild accessions that affect a set of interrelated biological processes, particularly the cell cycle. We postulate that such differences in expression profiles could partially explain the large differences in fruit sizes between domesticated and wild chili pepper varieties.

## 2. Results

We constructed and analyzed biological replicates of RNA-Seq libraries from 10 accessions (six domesticated (D) and four wild (W)), sampling every 10 DAA until maturity (see Materials and Methods and Supplementary S-1). Table 1 shows the accession, type, key, and time at which full maturity was reached in DAA.

A total of 22,427 genes, representing approximately 64% of the genes annotated in the *Capsicum* genome, were consistently expressed during fruit development in all of the accessions analyzed.

Gene expression varies by orders of magnitude [42], making it essential to perform data normalization [43], while differential gene expression allows one to contrast the relative expression of each gene under different conditions [44]. However, our aim was to evaluate changes throughout the time of fruit development, i.e., changes in gene expression profiles. With this purpose, we improved the method in [7] and defined the “standardized expression profile” (SEP), which is a seven-dimensional vector formed by the means of expression at each time point (0, 10, 20, 30, 40, 50, and 60 DAA), and it has a mean of 0 and standard deviation of 1 (see Materials and Methods and Supplementary S-2). The use of SEPs allows statistical comparisons between genes—or groups of genes—to be made independently of the relative gene expression of each gene.

For each gene within each accession, we estimated SEPs and analyzed differences between the D and W genotypes. Gene expression changes in times larger than 60 DAA in the late-maturing accessions (Ancho San Luis (AS), California Wonder (CW), and Jalapeño Espinalteco (JE)) were analyzed separately.

### 2.1. Analysis of Late Maturing Times

As seen in the “Maturity (DAA)” column in Table 1, three D accessions—AS, CW, and JE—take more than 60 DAA to reach the fully ripe fruit state; 70 DAA for CW and JE and 80 DAA for AS.

The precise time alignment of the last phenological states in all accessions is questionable; thus, we decided to perform a full analysis of seven time points that were uniformly sampled in all accessions (0,10,20,⋯,60 DAA) with the SEP methodology and to independently evaluate gene expression changes in late stages in these three accessions. With this aim, we performed contrasts between the times 60 and 70 in CW and JE, as well as 60 and 70 plus 70 and 80 in AS. Only 633 (2.82%) genes in total were differentially expressed in one or more of the tests performed in these contrasts, with a false discovery rate (FDR) threshold of 0.01 [45].

Moreover, by performing tests for all SEPs between the normal (Criollo de Morelos 334 (CM334) (CM), Serrano Tampiqueño 74 (ST), and Zunla-1 (ZU)) and late-maturing (AS, CW, and JE) accessions, only 37 (0.16%) of these gene expression profiles were significantly different at 0.01 FDR. In addition, the analysis of the expression profiles for the gene coding for “capsanthin/capsorubin synthase” (protein XP_016577952.1), which is expressed only in chromoplasts in late maturing states in *Capsicum* [5,46,47], showed no significant differences (p=0.24) between the normal and late-maturing accessions, implying that the analysis of SEPs from the 10 accessions using times between 0 and 60 DAA was reasonable, and would not induce a strong bias in the results. Details of these analyses are presented in Supplementary S-3.

### 2.2. Domesticated (D) and Wild (W) Accessions Have Different Mean SEPs

**Figure 1:**
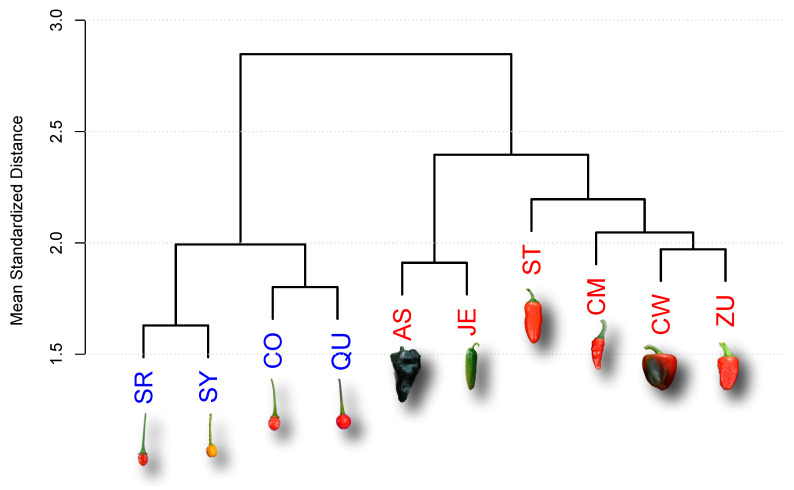
Dendrogram for the domesticated (D, red) and wild (W, blue) accessions. The dendrogram was obtained from the Euclidean distances between the full set of 22,427 standardized expression profiles (SEPs) of genes expressed during fruit development. Representative miniature photographs illustrate the fruits of the corresponding accessions. See Table 1 for accession names. Photographs of fruits are not at the same scale.

To study similarities in gene expression profiles between accessions, we calculated the mean Euclidean distances between the SEPs for all 22,427 genes expressed during fruit development to generate a dendrogram (Figure 1).

The D and W accessions form two distinct cluster groups, with a mean normalized distance of 2.85 on the Y-axis (Figure 1). The four W accessions (in blue) form a cluster at a mean distance of 2, whereas the six D accessions form a cluster at a mean normalized distance of approximately 2.4 (Figure 1 and Supplementary S-4).

**Figure 2:**
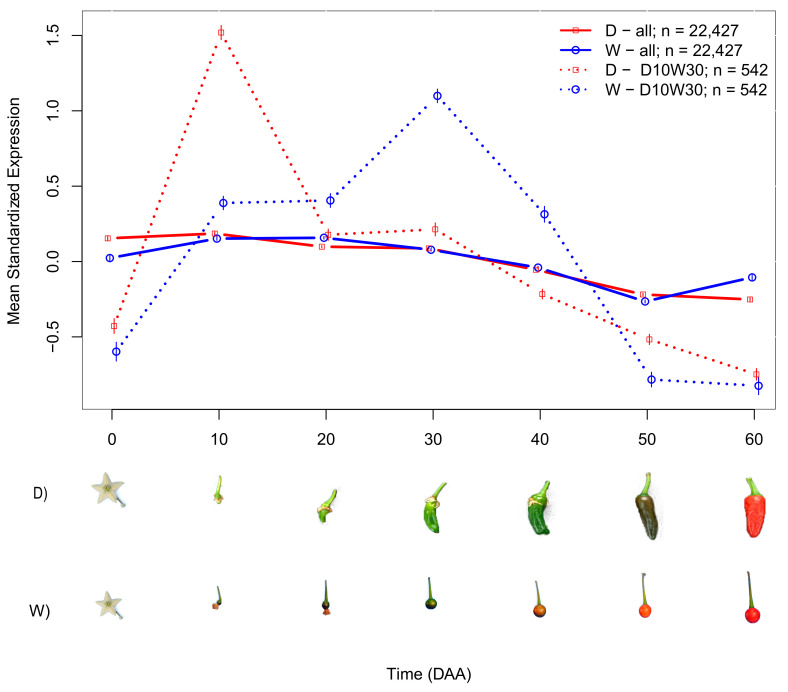
Average SEPs (Standardized Expression Profiles) for groups of genes in domesticated (D) and wild (W) accessions. Continuous colored lines (red and blue for domesticated (D) and wild (W), respectively) link the means of standardized gene expression at each time point for the complete set of expressed genes (*n* = 22,427). Dashed lines link the means of standardized gene expression at each time point for the *n* = 542 genes that presented the maximum expression at 10 DAA in D, while the maximum expression was reached at 30 DAA in W. These 542 genes form the group “D10W30” (see text). Representative miniature photographs illustrate the approximate fruit development of the D and W accessions at each time point. Photographs of fruits are not at the same scale.

To perform statistical analyses of genes or sets of genes, we considered contrasts between two groups of accessions: 6 D (AS, CW, JE, ST, and ZU in Table 1) and 4 W (Piquín Coahuila (CO), Piquín Queretaro (QU), Piquín Sonora Red (SR), and Piquín Sonora Yellow (SY) in Table 1). In all cases, the null hypothesis was that at each time point, the mean expressions of the D and W groups were equal, whereas the alternative was that these parameters differed. A t-test was used to obtain confidence intervals (CIs) for the means and to evaluate the significance at each of the seven time points sampled. We determined the mean SEPs for different gene groups in the D and W accessions (Figure 2).

The means for the D and W groups differed significantly (continuous line in Figure 2). At the mature flower state (0 DAA), the standardized mean expression for D was much higher than for W, implying that the average transcription activity in this state is substantially larger for the D genotypes. In the interval between 0 and 10 DAA, the mean standardized expression increased for both groups, although the rate of increase was higher for D. At 10 DAA, the mean expression for D reached a peak value, but for W, the increase continued, although at a slower rate, to peak at 20 DAA. From the peak at 10 DAA, the mean expression for D decreased at different rates, and was lower at all subsequent time points. The lowest value was seen at 60 DAA. In contrast, decreases in the mean expression for W began later, occurring from 20 up to 50 DAA, and reached a minimum of −0.27, which is smaller than the minimum for the D group, −0.25, seen at 60 DAA. The more relevant differences in the mean expression profiles between D and W were seen during the intervals 10 to 20 and 50 to 60 DAA, when the trend was inverted such that D was decreasing while W was increasing. On the other hand, less marked differences between D and W were seen between 30 and 50 DAA, when the mean standardized expression decreased nearly in parallel for both groups. The average of the time at which the maximum expression was reached in each group was five days earlier for D than for W. All observed differences were significant (see Supplementary S-4).

### 2.3. Differences in SEPs of Individual Genes Varied between D and W

A total of 463 genes, representing approximately 2.06% of the total, showed significant differences between D and W, with an FDR threshold of 0.05, which, for the individual tests, corresponds to a *p* value <0.000002. The differences in expression profiles between D and W were well defined and significant; the peak of the mean expression for D occurred at 10 DAA, while the peak for W occurred later at 30 DAA. The average times of maximum expression were 11.06 DAA for D and 28.33 DAA for W, or a difference of −17.27 DAA. Of the 463 selected genes, 36 (36/463≈0.08 or 8%) are transcription factors (TFs). This percentage is higher than that for TFs annotated in the *Capsicum* genome (1859/34,986≈0.05 or 5%). A list and description of the 463 selected genes and details of statistical analyses are presented in the Supplementary files SG and S-5, respectively.

We established that the main differences in SEPs between D and W were due to a set of 542 genes that presented an expression peak at 10 DAA in D, while the expression peak was at 30 DAA in the W accessions; we named these groups of genes “D10W30” (dashed line in Figure 2).

The results of this experiment showed differences in expression profiles between D and W at the levels of entire gene sets, groups of particular genes, and individual genes (see Supplementary S-5). Taking these findings together, we can thus conclude that there are relevant differences in expression profiles between domesticated and wild varieties of chili peppers during fruit development. We also found that gene expression diversity, expressed as the coefficient of variation of gene expression, is significantly (p=0.002) smaller in the D than in the W accessions, corroborating the findings presented by [48] for different species of plants and animals.

### 2.4. Differences in Expression of Genes Related to Cell Reproduction Appear Earlier and Are Larger in Domesticated Than in Wild Genotypes

Based on the evidence that mean SEPs differ between the D and W accessions, we investigated differences in the expression profiles in groups of genes related to particular biological processes. We first examined the mean SEPs of a group of 1125 genes associated with cell reproduction (Supplementary S-5.1). The mean expression value for 235 genes that are directly annotated in the cell cycle, but not in other cell reproduction processes, was significantly higher and occurred earlier for D compared to W, as evidenced by the peak of 0.3 standardized units at 10 DAA for D and 0.2 standardized units 30 DAA for W (Supplementary S-5.1). Similarly, the mean expression for 69 kinesins or kinesin-related proteins among the 1125 genes associated with cell reproduction exhibited a differential expression peak at 10 DAA for the D accessions, but for the W accessions, the peak was later at 30 DAA (Supplementary S-5.1). Thus, changes in the expression of genes associated with cell reproduction were significantly larger and occurred earlier for the D relative to the W accessions, not only for the full set of genes, but also for particular bioprocesses and gene families (Supplementary S-5–S-7).

### 2.5. Biological Processes Were Enriched in Genes That Are Expressed Earlier in Domesticated Genotypes

The results presented before indicate that the SEPs in the D and W accessions undoubtedly differ (Figure 2), and the genes for which expression peaks at 10 DAA for D but at 30 DAA for W (denoted here as “D10W30”) play an important role in cell reproduction. To validate and expand our study, we considered the D10W30 expression pattern in a Gene Ontology enrichment analysis (for details, see Supplementary S-5.2, S-6, S-7, and SG).

A total of 86 biological processes (BPs) were significantly enriched (FDR = 0.05; p<0.0015) in the D10W30 set, with a median odds ratio of 9.5. As such, these genes were much more abundant in these BPs than would be expected by chance. Apart from the aforementioned BPs related to cell reproduction, 43 of the enriched BPs, or 50% of the total, are involved in either positive or negative regulation of various biological processes. Of these, four (5%) are related to cellular component organization or biogenesis, three are associated with cellular component assembly, and another three play roles in organelle organization or fission. The general bioprocess, “cellular process” (GO:0009987), is also highly enriched in the D10W30 gene set, with an odds estimate of 2.25 and a highly significant *p*-value of $2.76\times 10^{-8}$.

These results show that genes with the pattern D10W30 are over-represented in important BPs, which, in turn, implies that the expression of such BPs occurs earlier and at higher levels in the D compared to the W genotypes.

Interesting examples of D10W30 genes involved in cell reproduction are the high-mobility group B protein 6 (XP_016555757.1), the MYB-related protein 3R-1 (XP_016537977.1), and the kinetochore protein NDC80 (XP_016539151.1); see Supplementary S-5.2 for the SEP plots. The gene encoding the “high-mobility group B protein 6” is a *WRKY* transcription factor involved in the nucleosome/chromatin assembly that was annotated in 12 of the 86 aforementioned BPs—particularly the cell reproduction BP. The gene encoding the transcription factor “MYB-related protein 3R-1” was included in 6 of the 86 enriched BPs and is mainly related to cellular, chromosome, and organelle organization. The “kinetochore protein NDC80” is part of the multiprotein kinetochore complexes that couple eukaryotic chromosomes to the mitotic spindle to ensure proper chromosome segregation. NDC80 is part of the outer kinetochore and forms a heterotetramer with the proteins NUF2, SPC25, and SPC24 [49,50]. Interestingly, the genes encoding NUF2 and SPC25 also exhibit the D10W30 expression pattern. NDC80 is conspicuously present in 74 of the 86 enriched BPs.

### 2.6. A Network of Cell Cycle Genes with the D10W30 Expression Pattern

**Figure 3:**
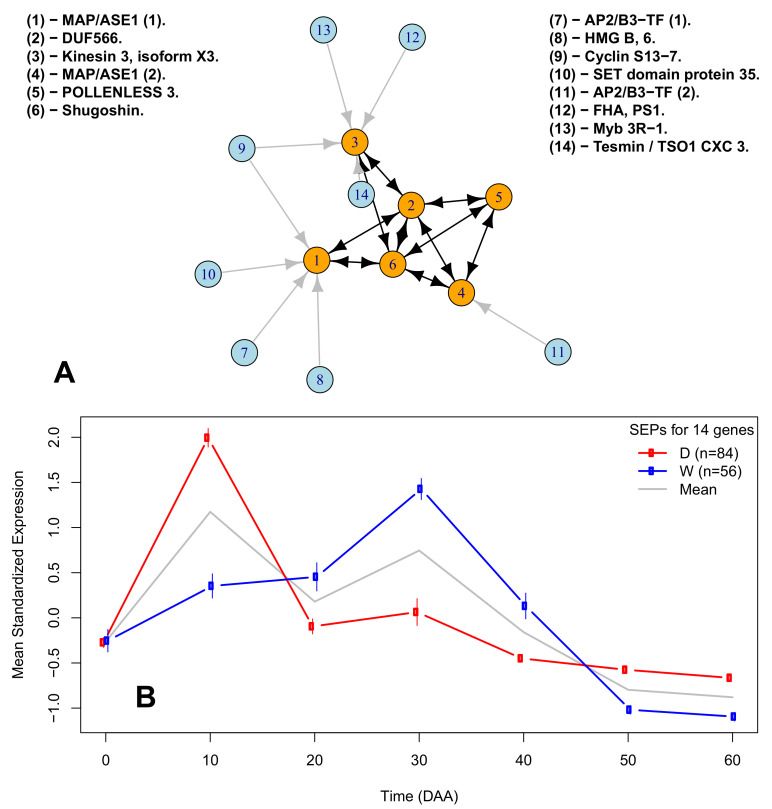
Gene network of the D10W30 cell cycle genes in the D and W accessions. (**A**) Graphic representation of the network. Orange circles represent structural genes, while blue ones represent transcription factor (TF) candidates for regulating the structural genes represented by orange circles (see Table 2 and Supplementary S-9). (**B**) Mean standardized expression of the genes in the network in the D and W accessions.

The availability of genome-wide gene expression technologies, as RNA-Seq, make it possible to identify gene interactions and represent them as gene networks [51]. In functional genomics, it is axiomatic that genes with highly similar expression profiles are likely to be regulated via the same mechanisms, and this hypothesis is the basis for the discovery of regulatory networks [52]. To show the concerted co-variation through time of cell cycle genes’ expression during fruit development, we estimated a gene network comprising six structural genes and eight TF candidates for regulating three of the six structural genes. Figure 3 presents the estimated gene network, while Table 2 gives the descriptions of the genes involved (Supplementary S-8–S-10).

Figure 3A presents the network formed by 14 D10W30 genes, in which the orange circles represent six structural genes involved in the cell cycle, and blue circles represent the TF gene candidates for regulating three of the structural genes (see the legend of Figure 3A and Table 2 for gene descriptions). Arrows between structural genes are drawn for genes with highly significant (p<0.0001) positive Pearson’s correlation coefficients (r>0.96) between mean SEPs within the D and W accessions, while Pearson’s correlation coefficients of the same gene pairs between the D and W accessions were small (r<0.4) and not significant (p>0.5). The origin and robustness of the network presented in Figure 3A can be appreciated by observing Figure 3B, which presents the mean SEPs for the 14 genes involved, which represent n=84 independent SEPs estimated from the six D accessions (red line; 84/6=14 genes) and n=56 independent SEPs estimated from the four W accessions (blue line; 56/4=14 genes). In Figure 3B, the 95% CI for the mean standardized expression of the genes—vertical lines at each time point—shows that these 14 genes are highly correlated within the D and W accessions, but have a very low correlation between the D and W groups. For details, see Supplementary S-8 and S-9.

**Figure 4:**
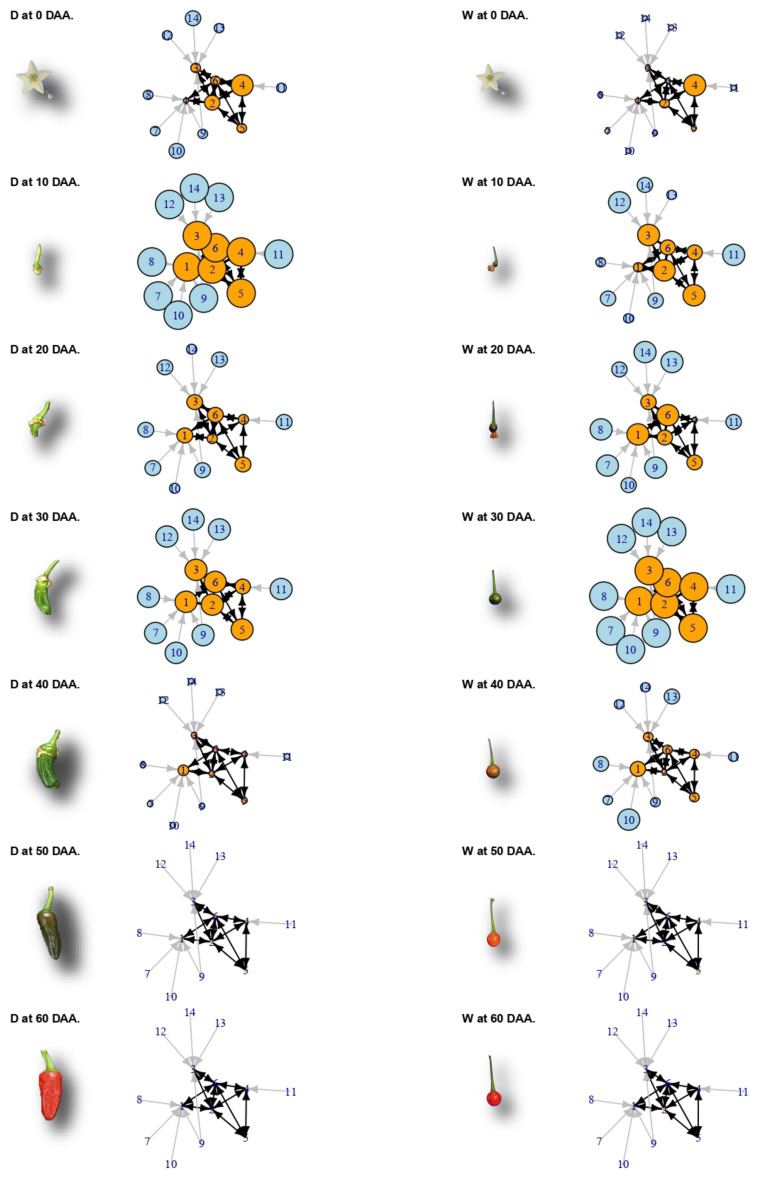
Change over time (rows) and groups of accessions (columns) of the network in Figure 3A. Gene expression is proportional to circle size. Photographs of fruits are not at the same scale.

For a better appreciation of the changes that occur in the individual standardized expression of the genes included in the network of Figure 3, Figure 4 presents a grid of the time and the accession groups, but in this case, the sizes of the circles representing genes vary in proportion to their individual standardized expression over time (rows; from 0 to 60 DAA) and sets of accessions (columns; D on the left-hand side and W on the right -hand side). Representative miniature photographs show the approximate fruit development stage for the D and W accessions.

In Figure 4, we can appreciate that individual gene expression is highly coordinated through time, but largely differs between the D and W groups of accessions. At 10 DAA in the D accessions (second row, left-hand side), all 14 genes have reached their maximum expression (largest circle sizes), while such expression levels are only reached 20 days later—at 30 DAA—for the W accessions (fourth row, right-hand side). Thus, the main cell cycle genes are well behind in maximum expression time in the W compared with the D accessions. In addition, expression changes occur faster in the D compared with the W accessions, a fact that can be verified by observing Figure 3B, where the slope of the line linking 0 and 10 DAA is more pronounced than the corresponding change to reach the maximum from 20 to 30 DAA in the W accessions. Departing from comparable standardized expression at the mature flower stage (0 DAA), individual expression rapidly diverges between the D and W groups to finally converge again to basal expression levels at 50 and 60 DAA. The concerted but highly divergent expression of cell cycle genes demonstrates that domestication has tailored this process to differ between the D and W genotypes, explaining, in part, the large differences in fruit size between these groups.

## 3. Discussion

Building on our previously described method for estimating the dynamics of the chili pepper transcriptome [7], as well as our time-course experiment [53] and statistical analysis methods, in this study, we generated entire gene expression profiles (SEPs) across the full course of fruit development to examine differential gene expression patterns between domesticated (D) and wild (W) varieties of chili peppers (Figure 1).

Domestication, as initially studied by Darwin [54], is currently defined as a distinctive coevolutionary and mutualistic relationship between domesticator and domesticate, and marked a key transition in human history [20]. Here, we propose that the differences observed between gene expression profiles in sets of domesticated (D) and wild (W) accessions (Figure 2) can be attributed to domestication.

Gene expression is an intrinsically noisy process [55]; however, our experimental design systematically took into account variations between sets of fruits by examining two replicates of each accession at each time point, as well as variation within the target groups (D and W), by examining six and four genotypes for each group, respectively. Thus, the differences observed between the D and W gene expression profiles can be attributed to differences in the selection histories of the two groups, i.e., to domestication. The effect of domestication on gene expression patterns is also corroborated by the fact that the D and W accessions belong to well-segregated clusters in the dendrogram presented in Figure 1. Although the distance between the D and W groups is approximately 2.85, the maximum distance within the groups is only 2.4, again demonstrating that gene expression patterns during fruit development were modified by domestication.

Selection during domestication can alter molecular footprints at the genomic level. For example, in *Capsicum*, Qin et al. [36] identified 115 genomic regions containing 511 genes that show strong selective sweep signals due to domestication. The same method of searching for selective sweep signals identified candidate genes not only in plants, such as maize [23], sunflower [56], soybean [57], and Asian rice [58], but also in domesticated animals, such as dogs [59] and cattle [60]. Ross-Ibarra et al. [21] reviewed methods of identifying the genes responsible for adaptation to domestication and classified them in terms of the phenotype–genotype hierarchy as “top-down”, in which the method starts with a phenotype to identify candidate genes, and as “bottom-up”, in which genetic analyses are used to identify adaptive genes. Bioinformatics tools are then used to connect the selected genes to a phenotype. Our approach in this study can be considered as a hybrid between “top-down” and “bottom-up” methods in that we began with a molecular phenotype, i.e., a standardized gene expression profile (SEP), which showed that there are significant differences between the D and W groups of accessions, and then examined the biological relevance of these findings.

Expression levels of genes that are important during chili pepper fruit development have been reported in [11,12,13]. Differences in the accessions employed, times of development, genes analyzed, and methodologies preclude full and equitable comparisons between the results presented in those references with the ones presented here. Examination of 74 SEP plots for genes studied in the mentioned works resulted in coincidences of 62% with our results. This percentage can be considered to be high, given the dissimilarities of the above-mentioned factors (plots and results available upon request). On the other hand, Díaz-Valenzuela et al. [41] reported results from the analyses of one cultivated accession, one wild accession, and their $F_{1}$ only at 40 DAA. Given that this study does not include gene expression measurements at different times of fruit development, it is impossible to examine their findings in the framework of our work.

Differences in gene expression between crops and their wild ancestors were reported in tomato [29,61], which, like *Capsicum*, belongs to the Solanaceae family, as well as common bean [26,27], carrot [62], ramie [63], and cotton [64]. For animals, similar comparisons were done for turkey [65], trout [66], and other species [67]. All of these studies reported sets of differentially expressed genes between the domesticated and wild forms, but only for tomato did the study authors use a time-course experiment to evaluate the deceleration of the circadian clock [29]. On the other hand, a decrease in gene expression diversity in domesticated forms of a set of animals and plants was reported by Liu et al. [48]. Analysis of our data confirmed that gene expression is significantly less diverse in domesticated chili peppers compared to that seen for wild accessions.

Normalization of the gene expression profiles of genes or sets of genes—discussed here as mean SEPs (see Results) —implies that the mean over time of the profile is equal to zero, and the standard deviation is equal to one. This transformation allows direct and unbiased comparisons of expression profiles in the D and W groups of accessions, with a statistical evaluation performed at each time point (Figure 2 and Supplementary S-4 and S-5).

It is important to note that the differences in mean SEPs between D and W were produced by a set of genes that were likely affected by the domestication process (Figure 2). However, the large majority (21,666/22,427;96.6%) of expressed genes did not exhibit significant differences in the mean expression profile (Supplementary S-5), implying that a large part of the transcriptome during fruit development was not affected by domestication.

In chili pepper, as in tomato, the first ten days after anthesis (DAA) are characterized by a period of very active cell division, in which the number of cell layers across the pericarp doubles compared with that at anthesis, and this number is maintained through the end of development [68]. Fruit growth then proceeds into the cell expansion phase and continues until ≈40 DAA, progressing to full ripening at ≈50 DAA before entering senescence at ≈60 DAA [7]. During the period of cell expansion—from approximately 10 to 40 DAA—the dominant process is cell endoreduplication [68,69,70]. In *Capsicum*, pericarp thickness has a high positive correlation with the degree of polysomaty [71], which implies that accessions that have a thick pericarp have higher endoreduplication compared to accessions with a thin pericarp. This relationship has been corroborated through the induction of different ploidy levels [72].

The time lag observed for the peak of mean standardized expression between the D and W accessions across the entire set of expressed genes (Figure 2) implies that domestication caused a shift in the time and intensity of gene expression, favoring an earlier and higher expression maximum in D. Considering 463 genes that had the largest differences in expression profiles between D and W confirmed that the peak of expression for D occurs at 10 DAA compared to 20 DAA for W, and the peak expression value is significantly higher for D than for W. A similar expression pattern is seen for a gene encoding a G2/mitotic-specific cyclin, which is essential for control of the cell cycle at the G2/mitosis transition (Supplementary S-5). The transcript of this gene accumulated steadily during G2 and abruptly declined at mitosis. In *Arabidopsis*, which exhibits a similar trend in expression, members of the cyclin family are thought to be part of a developmental mechanism that coordinates the switch between proliferation and endoreduplication [73].

Here, we found the peak gene expression for D at 10 DAA, a time when cell division is very active [68]. Furthermore, domesticated accessions bear substantially larger fruits than wild accessions [33], and this larger fruit size is primarily achieved through increases in cell numbers [74]. As such, we grouped a set of 1125 genes associated with cell reproduction by including genes annotated in nine bioprocesses and examined the gene expression profiles (Supplementary S-5). Genes annotated for the cell cycle presented an earlier increase and higher mean expression in D relative to W (Supplementary S-5). Given that both cell number and cell size, which are respectively determined by cell division and cell expansion [75], contribute to fruit size, it is compelling that, in the D accessions, the cell division expression profile peaked earlier and higher than in the W genotypes. This finding is consistent with that seen for tomato, in which cell division genes strongly influence fruit yield [76].

The mean expression of the genes related to the cell cycle presented a profile characterized by a large peak expression at 10 DAA for D, whereas the W accessions had a smaller peak that occurred later at 30 DAA. We isolated a set of 542 genes that presented this pattern, which we termed “D10W30”. Gene Ontology enrichment analyses produced a set of 86 biological processes (BPs) that were highly enriched among the D10W30 genes (Supplementary S-6 and SG). In addition to four cell reproduction BPs, this set included 43 BPs associated with regulation of different cell processes, including negative regulation of cellular process, as well as protein modification and transferase activity. Interestingly, such negative regulation has been associated with fruit development and ripening [77]. Other selected BPs include four that are related to cellular component organization, which has been linked to the accumulation of soluble sugars and organic acids in fruits [78], and three that are related to cellular component assembly and also showed differential expression in a proteomic study of *Capsicum* [79]. Another three in this set were identified with organelle fission or organization, and two were related to microtubule-based processes or movement, and are clearly associated with mitosis; they have been linked to floral development in the genus *Aquilegia* [80] and to the autophagy BP, which allows remodeling of intracellular structures during cell differentiation [81]. In *Arabidopsis*, this BP has been linked with the complete proteolysis of stromal proteins [82]; see Supplementary S-6, S-7, and SG for details.

We plotted the expression profiles of genes that follow the D10W30 pattern, i.e., expression peaks at 10 and 30 DAA for the D and W accessions, respectively (Figure 2). Interesting examples of this expression pattern are genes encoding: (i) the high-mobility group B protein 6 (HMG B, 6), which belongs to a group of chromosomal proteins that regulate DNA-dependent processes and display a highly dynamic nuclear localization [83]; (ii) the transcription factor “MYB-related protein 3R-1”, which, in *Arabidopsis*, synergistically maintains G2/M-specific genes repressed in post-mitotic cells and restricts the time window of mitotic gene expression in proliferating cells, thus giving it a role in determining organ size [84]; and (iii) the kinetochore protein NDC80, which is an essential component of the kinetochore complex that mediates chromosome segregation and spindle checkpoint activity to ensure proper cell division. In *Arabidopsis*, the NDC80 mutant *mun-1* has a reduced cell division rate, aneuploidy, and defects in chromosome segregation [85]; see Figure 3 and Supplementary S-5.

The D10W30 set includes genes coding for “microtubule-associated protein TORTIFOLIA1” and the “protein TPX2”, which have genomic fingerprints for domestication in *Capsicum* [36]. TORTIFOLIA1 (TOR1) is a plant-specific microtubule-associated protein (MAP) that regulates cortical microtubule orientation and the direction of organ growth [86]. TOR1 also determines microtubule organization by modulating microtubule severing [87] and participates in organ elongation [88]. On the other hand, TPX2 performs multiple roles in microtubule organization [89], such as regulating prospindle assembly before nuclear envelope breakdown [90], and is linked to fruit development in European pear [91]. Of the 300 domestication genes reported by Qin et al. [36] and expressed during fruit development, 59 (≈20%) also showed significant (p<0.05) differences between the D and W accessions in this study.

An example of the coordinated time lag existent between the D and W accessions is presented in Figure 3 and Figure 4. The 14 genes involved in this network are pivotal for the cell cycle process and are thus functionally related, but also present highly coordinated gene expression profiles within the D and W accessions, which markedly differ between these two groups by having the D10W30 expression pattern (Supplementary S-8, S-9, and S-10). It is important to assess the robustness of the links inferred between the genes in this network (Figure 3). With this aim, we must take into account the fact that such links were inferred from ten fully independent datasets, i.e., the ones corresponding to each one of the 10 accessions. Then, if we assume that the selection of a gene to be part of the network has an error probability “ε”, then the probability of erroneously selecting a gene to be part of the network repeatedly in the ten accessions is $\varepsilon^{-10}$. Thus, even if the individual error probability is large—for example, ε=0.1—the probability of having committed such errors repeatedly in the ten accessions is vanishingly small; for ε=0.1, this equals $10^{-10}$, or one in ten billion.

Of the genes involved in the network of Figure 3A, two of them—(1) and (4) in the figure legend—encode two versions of the microtubule-associated protein 3. The *Arabidopsis* orthologous of these genes, PLEIADE/AtMAP65-3, has been shown to have physical and genetic interactions with the Transport Protein Particle II (TRAPPII), which is required for coordinating cytokinesis with the nuclear division cycle [92]. Gene (2) in Figure 3A, labeled as *DUF566* in the legend, contains the InterPro domain IPR007573 and corresponds with the *Arabidopsis* orthologous *AT2G44190* (Table 2), which codes for the endosperm-defective 1 (*ede1*) gene; this encodes a microtubule-associated protein that is essential for microtubule function during the mitotic and cytokinetic stages, which generate the endosperm and embryo, and is thus essential for seed formation [93]. Gene (3) in Figure 3 encodes a kinesin 3, which is an *Arabidopsis* ortholog, *AT4G21270*, and encodes the *ATK1* gene, which has been demonstrated to be required for spindle morphogenesis [94]. Consistently, the large majority of chili pepper kinesins follow the D10W30 expression pattern (Supplementary S-5.1). The gene labeled as “(5)—POLLENLESS 3” in the legend of Figure 3A contains a tetratricopeptide repeat (TPR), and its closest *Arabidopsis* ortholog, *AT4G20900*, encodes the TDM1 gene, which has been previously shown to be essential for meiotic termination [95]. Structural gene (6) in Figure 3A encodes shugoshin, a conserved kinetochore protein that prevents dissociation of cohesin from centromeres during mitosis [96]. The closest *Arabidopsis* ortholog locus of this chili pepper sequence, *AT3G44960*, encodes five splicing variants of shugoshin, one of which has been reported to protect centromeric cohesion during meiosis [97]; see also Supplementary S-10. It is intriguing that three of the chili pepper genes, the ones labeled as numbers (3), (5), and (6) in the legend of Figure 3A, have as their closest orthologs in Arabidopsis genes that are reported primarily in meiosis [94,95,97], respectively, while the expression patterns of these *Capsicum* genes clearly correspond to genes involved in mitosis. This fact could be due to the large functional divergence between the *Capsicum* and *Arabidopsis* genomes, which diverged from a common ancestor more than 150 million years before present [36].

To select TF candidates for regulating the expression of the structural genes, represented by orange circles in the network of Figure 3, we employed an algorithm for TF imputation, which is detailed in Supplementary S-9. This algorithm relies on stringent thresholds for correlations in expression estimated for each accession, and has a very low probability of false-negative imputations, estimated in this case as $1.06\times 10^{-15}$. The algorithm was successful in recovering a TF that was experimentally confirmed to be regulating the expression of the *AT3* gene in *Capsicum* [8,98,99] (see Methods and Supplementary S-9).

In summary, the functional network presented in Figure 3 and Figure 4 demonstrates that domestication has produced a time lag in the expression of core cell cycle genes, thus anticipating by approximately 20 days the maximum expression of those genes and producing a higher standardized expression at the early fruit development stage (10 DAA) in D when compared with the W accessions.

Comparing gene expression profiles rather than focusing on the differential expression of single genes at a given time gives a better perspective on the complex interplay occurring in the transcriptome over time. Here, we demonstrated that a set of genes exhibits significant differences in expression profiles between the D and W accessions during the development of chili pepper fruit. Genes in this set are associated with processes that involve the cell regulation, cycle, localization, motility, and assembly, as well as with autophagy and organelle organization. In particular, differences in the time and intensity of the expression of genes are related to cell reproduction and provide an explanation at a molecular level of differences in fruit size between the D and W accessions, which is the main morphological difference between these two genotype groups [100].

## 4. Materials and Methods

### 4.1. Statistical Design

RNA-Seq was performed as a factorial experiment with time (seven levels: 0, 10, 20, 30, 40, 50, and 60 DAA) and accession (10 accessions: 6 domesticated and 4 wild; see Table 1) as factors. Additional RNA-Seq libraries were constructed and analyzed for accessions with later maturation times: at 70 DAA for accessions CW and JE and at 70 and 80 DAA for AS. The RNA-Seq library was the experimental unit, and two libraries of every combination of time per accession were independently replicated two times; thus, we analyzed 7×10×2=140 RNA-Seq libraries for time points between 0 and 60 DAA in all 10 accessions—plus four libraries at 70 DAA in accessions CW and JE and four libraries corresponding to 70 and 80 DAA in AS—for a total of 140+4+4=148 RNA-Seq libraries. After quality control, the raw reads were mapped to the *Capsicum* genome (CM334 v1.6) to obtain reliable estimations of gene expression. The relative expression for each gene at each accession and time combination was considered here as the output variable.

### 4.2. Plant Materials and Cultivation

Seeds of each one of the D *Capsicum annuum* L. accessions (Table 1) were surface sterilized with a 70% ethanol solution for 10 s before treatment with a 10% hypochlorite solution for 10 s and six rinses with distilled water. Wild accession seeds were similarly treated after an initial treatment with 50% sulfuric acid solution to break seed dormancy. All accession seeds were germinated in plastic trays containing a mixture of three parts peat moss, one part perlite, one part vermiculite, one part sludge, and two parts forest soil in a growth chamber, with 16 h light (photon flux of 70 μmol m${}^{-2}$ s${}^{-1}$) at 28 ${}^{\circ }$C and 66% relative humidity. Three-week-old chili pepper plants were transplanted individually into 5 L plastic pots containing the same soil mixture as that described above. During transplantation, 15 g of a mixture of mycorrhizal fungi and beneficial bacteria was added to optimize root growth and development. Plants were fertilized with Long Ashton solution every two weeks. Flowers and fruits at each one of the times of development (0, 10, 20, 30, 40, 50, 60, 70, or 80 DAA, depending on the final maturation time; see Table 1) were collected, immediately frozen in liquid nitrogen, and stored at −80 ${}^{\circ }$C.

### 4.3. RNA-Seq Library Construction and Processing

Total RNA was extracted from flowers and whole chili pepper fruits at different developmental stages using a NucleoSpin${}^{\mathrm{TM}}$ RNA Plant kit (MACHEREY-NAGEL) according to the manufacturer’s instructions. RNA was extracted from two biological samples comprising either flowers or fruits from 2–6 different plants. RNA quality was verified by determining the RNA integrity number (RIN) for each sample (Supplementary S-1). Samples of total RNA were shipped to Novogene (https://en.novogene.com/ accessed on: 17 March 2021) for library construction, sequencing, and mapping to a reference genome. At Novogene, libraries were prepared and sequenced using the Illumina NovaSeq platform to obtain at least 20 million raw paired-end reads of 150 bp per sample. These reads were subjected to quality control and then mapped to the *Capsicum* reference genome CM334 v1.6 (http://peppergenome.snu.ac.kr/ accessed on: 17 March 2021). Novogene provided the matrix of raw counts per library for each of the 35,883 *Capsicum* genes. These genes were identified by a protein product (when known) and annotated with Gene Ontology (GO) and Kyoto Encyclopedia of Genes and Genomes (KEGG) terms. For this study, a total 140 libraries were processed, yielding more than 2.29 billions of clean reads mapped to the genome. The number of reads mapped to the genome had a minimum of 10.33, a median of 16.26, a mean of 16.42, and a maximum of 23.86 million reads per library; see Supplementary S-1 and SG.

### 4.4. Estimations and Analyses of Standardized Expression Profiles (SEPs)

To avoid inclusion of genes that had very low or inconsistent expression patterns, only genes that had a raw count of >0 in at least two of the replicates per accession were selected for analysis. After this filtering, 22,427 genes remained for analyses, and of these, ≈62.5% were identified in the *Capsicum* genome; see Supplementary SG.

All results were maintained in an in-site MySQL (https://www.mysql.com/ accessed on: 17 March 2021) relational database, and were analyzed with R version 3.4.4 [101]. For each of the 10 accessions, we used the R package “edgeR” version 3.20.9 [44] to obtain *p* values for each gene in each accession in all contrasts between neighbor time intervals, i.e., 0–10, 10–20, 20–30, ⋯, 50–60 DAA. Following the method presented in [7], the gene expression tendency at each interval was classified as decreasing, steady, or increasing, with adjustment of the Type I error to 0.01. For each gene within each accession, the mean standardized expression was calculated, and the resulting seven-dimensional vector constituted the standardized expression profile (SEP) in downstream analyses. There were 10 SEPs for each of the 22,427 genes that were consistently expressed during fruit development, with one for each of the accessions, and these SEPs were classified into the groups of interest: domesticated (D) with six elements and wild (W) with four elements. To evaluate the difference between the D and W SEPs, we calculated the Euclidean distances between and within the groups and tested the hypothesis of equality of distances between and within SEPs with a t-test. Using an FDR of 1% (i.e., a *q* value ≤0.01), genes were classified as having an equal or distinct SEP in the D and W groups. For individual genes or groups of genes, we calculated the 95% CI for the means at each of the seven time points sampled, as well as the *P* value for the t-test of mean equality. A set of R functions was programmed to data-mine the results. The statistical procedures are described in detail in Supplementary S-2 to S-9, and a manuscript presenting the R package for data-mining the results is under evaluation [102].

### 4.5. Network Estimation

We began the network estimation with the 25 genes with expression profile D10W30, which was annotated with the cell cycle biological process in the 10 accessions. For all 25×(25−1)/2=300 different pairs formed by these 25 genes, we calculated the Euclidean distance between their mean SEPs, selecting the pairs with a distance of less than 1 standardized units. This stringent criterion selected only 10 gene pairs (3.3% of the total, and shown as edges between orange circles in Figure 3A), which included the six structural genes (orange circles in Figure 3A). The SEPs of the 10 gene pairs linked with black double-headed arrows have a large (r>0.96) and highly significant (p<0.0001) mean Pearson’s correlation within the D or W genotypes. In contrast, the corresponding mean correlation between the D and W accessions was small (r<0.4) and not significant (p>0.5), demonstrating that while these genes have a highly concordant expression within the D and W groups, the expression profiles are very different between those groups (Figure 3B and Supplementary S-8). Finally, the selection of the 8 TF candidates for regulating the expression of the structural genes in the network of Figure 3A was performed by employing an algorithm that selected TFs with a highly concordant expression profile between TFs and each target gene (see Supplementary S-9 for details).

## Figures and Tables

**Table 1:** Chili pepper accessions used in this study.

Accession	Type	Key	Maturity (DAA)
Ancho San Luis	D	AS	80
Criollo de Morelos 334 (CM334)	D	CM	60
California Wonder	D	CW	70
Jalapeño Espinalteco	D	JE	70
Serrano Tampiqueño 74	D	ST	60
Zunla-1	D	ZU	60
Piquín Coahuila	W	CO	60
Piquín Queretaro	W	QU	60
Piquín Sonora Red	W	SR	60
Piquín Sonora Yellow	W	SY	60

**Table 2:** Identifiers and descriptions for genes in the network of Figure 3A.

Figure 3A Legend	Protein ID	Description	Ortholog
(1)-MAP/ASE1 (1).	XP_016564755.1	65-kDa microtubule-associated protein 3	AT5G51600
(2)-DUF566.	XP_016538322.1	ENDOSPERM DEFECTIVE 1 (DUF566)	AT2G44190
(3)-Kinesin 3, isoform X3.	XP_016541615.1	Kinesin 3 isoform X3	AT4G21270
(4)-MAP/ASE 1 (2).	XP_016575449.1	65-kDa microtubule-associated protein 3 isoform X1	AT5G51600
(5)-POLENLESS 3.	XP_016577799.1	Protein POLLENLESS 3	AT4G20900
(6)-Shugoshin.	XP_016548908.1	Shugoshin-1; chromosome segregation	AT3G44960
(7)-AP2/B3-TF (1).	XP_016568750.1	AP2/B3-like TF family protein	AT5G42700
(8)-HGM B, 6.	XP_016555757.1	High-mobility group B protein 6	AT4G11080
(9)-Cyclin S13-7.	XP_016543946.1	G2/mitotic-specific cyclin S13-7	AT3G11520
(10)-SET domain protein 35.	XP_016547461.1	SET domain; methyltransferase activity.	AT1G26760
(11)-AP2/B3-TF (2).	XP_016575946.1	AP2/B3-like TF family protein	AT5G58280
(12)-FHA, PS1.	XP_016574880.1	FHA domain-containing protein PS1	AT1G34355
(13)-Myb 3R-1.6	XP_016537977.1	Myb-related protein 3R-1	AT4G32730
(14)-Tesmin / TSO1 CX3 3.	XP_016565918.1	Protein tesmin/TSO1 CXC 3	AT3G22780

## Data Availability

The data discussed in this publication have been deposited in NCBI’s Gene Expression Omnibus [103] and are accessible through the GEO Series accession number GSE165448 (https://www.ncbi.nlm.nih.gov/geo/query/acc.cgi?acc=GSE165448 accessed on: 17 March 2021). Additionally, the R [101] package *Salsa* [104], which contains the data as well as functions for exploring the *Capsicum* transcriptome, is available at Zenodo (https://zenodo.org/record/4587745#.YEUDtbSuLaY accessed on: 17 March 2021).

## Supplementary Materials

The following are available online at https://www.mdpi.com/2223-7747/10/3/585/s1: Supplementary methods and results (Supplementary.pdf). Sections of this document are referred to in the text as ‘S-#’, where ‘#’ is the number of the corresponding section. Supplementary Excel file with four sheets, including information about genes and analyses of bioprocesses (SG.xlsx).

## Abbreviations

The following abbreviations are used in this manuscript:

D	Domesticated accession (Table 1)
W	Wild accession (Table 1)
DAA	Days after anthesis
SEP	Standardized expression profile
FDR	False discovery rate
