# Supplementary material for: Transcriptome Analyses Throughout Chili Pepper Fruit Development Reveal Novel Insights into the Domestication Process
Source: Plants (Basel). 2021 Mar 19;10(3):585. doi: 10.3390/plants10030585 (PMC8003350; doi:10.3390/plants10030585)
Supplement: Supplementary file 1 [file plants-10-00585-s001.zip › Supplementary.pdf]

SUPPLEMENTARY METHODS AND RESULTS FOR "Transcriptomic Analyses Throughout Chili Pepper Fruit Development Reveal Novel Insights into Domestication Process"

OCTAVIO MARTÍNEZ, MAGDA L. ARCE-RODRÍGUEZ, FERNANDO HERNÁNDEZ-GODÍNEZ, CHRISTIAN ESCOTO-SANDOVAL, FELIPE CERVANTES-HERNÁNDEZ, CORINA HAYANO-KANASHIRO, JOSÉ J. ORDAZ-ORTIZ, M. HUMBERTO REYES-VALDÉS, FERNANDO G. RAZO-MENDIVIL, ANA GARCÉS-CLAVER AND NEFTALÍ OCHOA-ALEJO.

Notes

- Sections of this document are cited in the main text of the paper as "S-#", where '#' corresponds to the section in the table of Contents (below).
- The data discussed in this publication have been deposited in NCBI's Gene Expression Omnibus (Edgar et al., 2002) and are accessible through GEO Series accession number GSE165448.
- Analyses presented here were performed in R (R Core Team, 2013) version 3.4.4, and can be reproduced using the R package "*Salsa*" Version 0.4. (Escoto-Sandoval et al., 2020; Martínez and Escoto-Sandoval, 2021).
- The "*Salsa*" R package (Salsa: An R package of data mining facilities for Capsicum gene expression profiles) can be downloaded from the link "Salsa at zenodo".
- In an effort to follow the standards of reproducible research (Peng, 2011), all relevant information is stored into a MySQL relational database named 'SALSA'. A dump of that file is available upon request.

Contents

S-1. Library sequencing and mapping to reference genome. 2
S-2. Standardized Expression Profile (SEP) estimation 6
S-3. Analysis of late maturing times 10
S-3.1. Gene tendencies during fruit development (from 0 to 60 DAA) 10
S-3.2. Gene tendencies in late accessions (AS, CW and JE) 11
S-3.3. SEP differences between 'normal' and 'late' accessions 13
S-4. Testing differences between Domesticated (D) and Wild (W) SEPs 14
S-5. Analyses per time of SEPs in D and W accessions 15
S-5.1. Differences in Expression of Genes Related to Cell Reproduction Appear Earlier and are Larger in Domesticated than Wild Genotypes 21
S-5.2. Biological Processes Enriched in Genes That Are Expressed Earlier in Domesticated Genotypes 23
S-6. Gene Ontology (GO) enrichment analyses 25
S-7. Genes and Bio Processes (BPs) reported. 25
S-8. Network estimation 27
S-9. Transcription Factor (TF) imputation 28
S-10. Supplementary descriptions and web links for genes in the network 30
References 33
S-11. Appendix (R output) 35
S-12. Analyses of gene with id=580 (FBN); see Figure 11 which presents the plot obtained with the function. 35

Date: March 12, 2021.

S-12.1. Analyses of gene with id= 19147 (B3 domain-containing protein); see Figure 13 which presents the plot obtained with the function. 36
S-12.2. Analyses of GO biological process "Cell Cycle" having as target the D10W30 set of genes. 37
S-13. Appendix (Clean reads per library and genotype) 39

S-1. Library sequencing and mapping to reference genome.

As mentioned in the main text, after extraction we shipped the total RNA samples to Novogene for quality control, sequencing and mapping to reference genome CM334 v1.6. Here we briefly describe and exemplify the procedures carried out in Novogene.

RNA sequencing was carried out in the Illumina NovaSeq platform, based on mechanism of SBS (sequencing by synthesis), and the sequencing workflow of the project is illustrated in Figure 1a, while Figure 1b shows the pipeline of the analyses and Figure 1c presents the quality control pipeline for the filtering of raw reads.

Original image data file from the Illumina sequencing platform were transformed into sequenced reads (raw reads) by CASAVA base recognition (Base Calling). Raw data are stored in FASTQ (fq) format files, which contain sequences of reads and corresponding base quality. In Figure 1c we see the post-processing of raw reads which consisted in (1) Remove reads with adaptor contamination, (2) Remove reads when uncertain nucleotides constitute more than 10 percent of either read (N $>$ 10%), (3) Remove reads when low quality nucleotides (Base Quality less than 20) constitute more than 50 percent of the read.

Figure 6b presents examples of the results obtained from the library for sample 'AS00R1' (Replicate 1 of the time 0 DAA from accession AS); for brevity not all results are shown for this library and there are results for a total of 140 libraries, all of which were visually inspected before further processing. Figure 2a presents the plot of percentage of error rate (Y-axis) by position along the reads (X-axis), and in general, a single base error rate should be lower than 1%. Figure 2c shows the reads distribution to the reference genome as percentage of total raw reads, in categories (1) Adaptor related: (reads containing adapter) / (total raw reads), (2) Containing N: (reads with more than 10% N) / (total raw reads), (3) Low quality: (reads of low quality) / (total raw reads) and (4) Clean reads: (clean reads) / (total raw reads). For all libraries the large majority of reads were in class (4), i.e., clean reads. Figures 2c and 2d refer to mapping the reads in the reference genome and will be commented below.

The algorithm for mapping filtered sequenced reads to the reference genome is shown in Figure 6c.

In Figure 6c shows how the program HISAT2 was run with default parameters to map the clean reads to the genome. As examples of the result of the process Figure 2c shows the reads distribution to the reference genome by categories while Figure 2d shows the reads densities in chromosomes, in both cases for a single library, 'AS00R1' (Replicate 1 of the time 0 DAA from accession AS).

A total of more than 2.29 billions of clean reads from the RNA-Seq libraries were mapped to the genome, and the number of clean reads reads mapped to the genome per library ranges from a minimum of 10.33 millions up to a maximum of 23.86 millions with a mean of 16.42 millions. The numbers of clean reads per library and genotype that were map to the reference genome are presented in Appendix S-13.

To evaluate the accuracy of the results as well as the efficiency of the experimental procedures we can use the matrix of correlation coefficients between gene expression in samples. Figure 6d shows a partial view of that matrix for only 56 of the 140 libraries.

Correlation of the gene expression levels between samples plays an important role to verify reliability and sample selection, which can not only demonstrate the repeatability of the experiment but estimate the differential gene expression analysis as well. The closer the correlation coefficient is to 1, the higher similarity the samples are. Encode suggests that the square of the Pearson correlation coefficient, r, should be larger than 0.92 (under ideal experiment conditions). Correlation coefficients between samples indicates that the expression pattern is closer. In Figure 6d higher correlation coefficients, r, are represented by

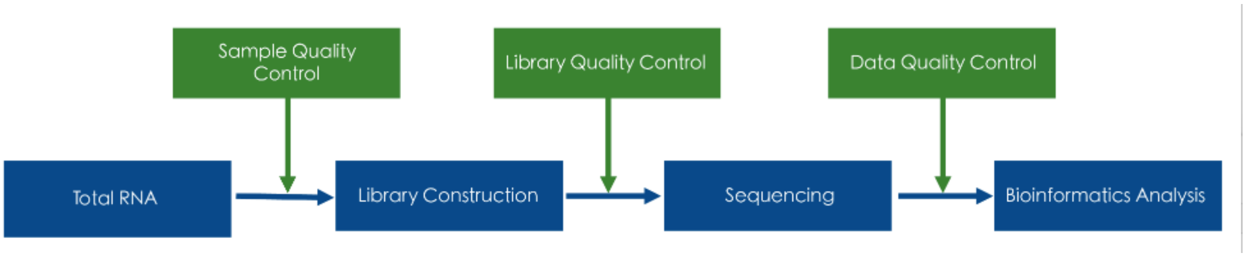

(A) RNA sequencing workflow

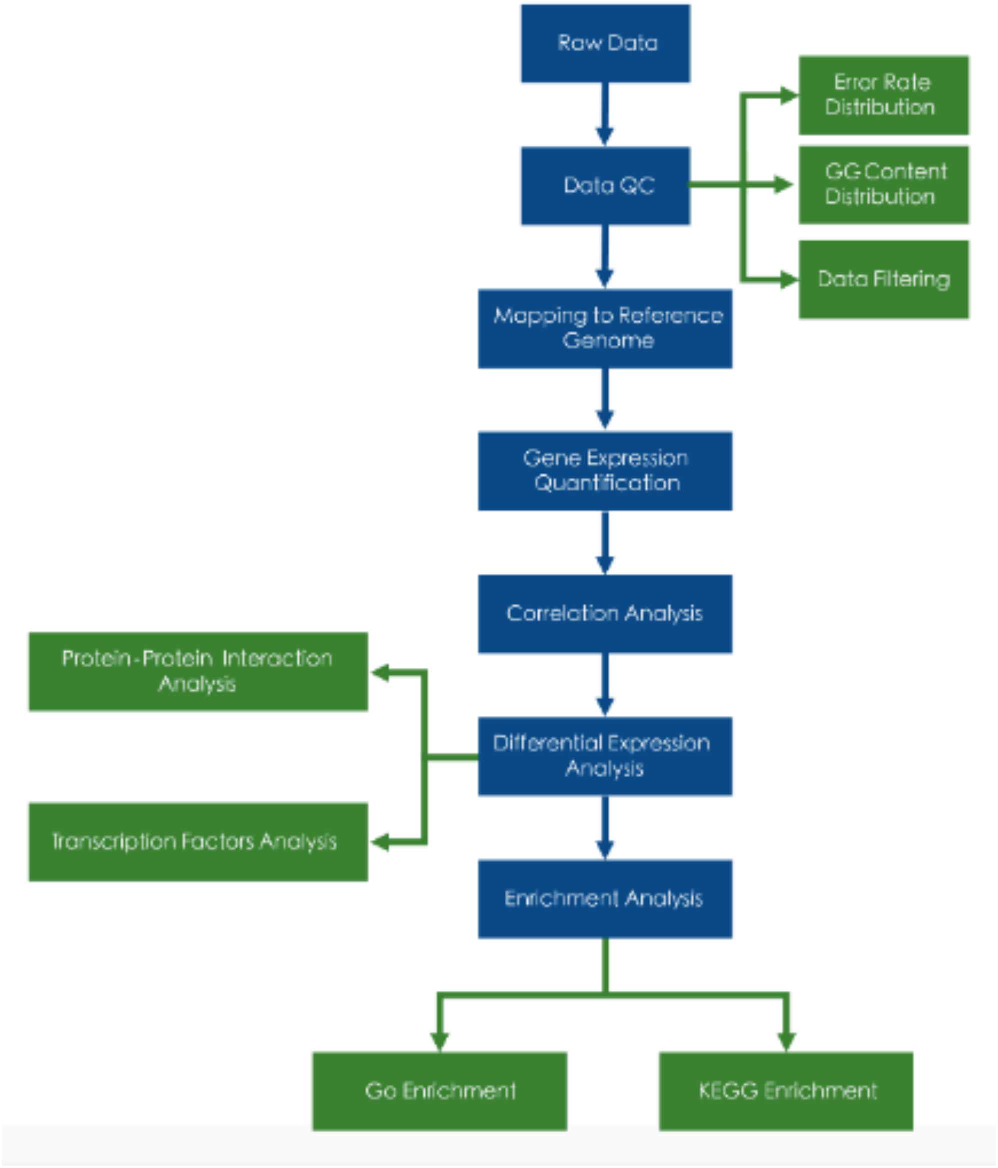

(B) Analysis Pipeline

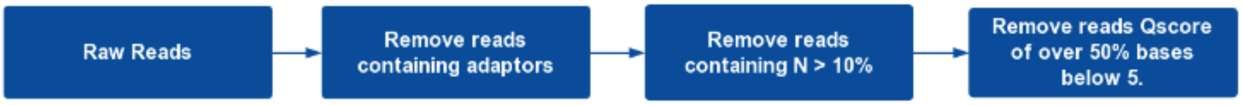

(C) Raw reads filtering

FIGURE 1. General procedure

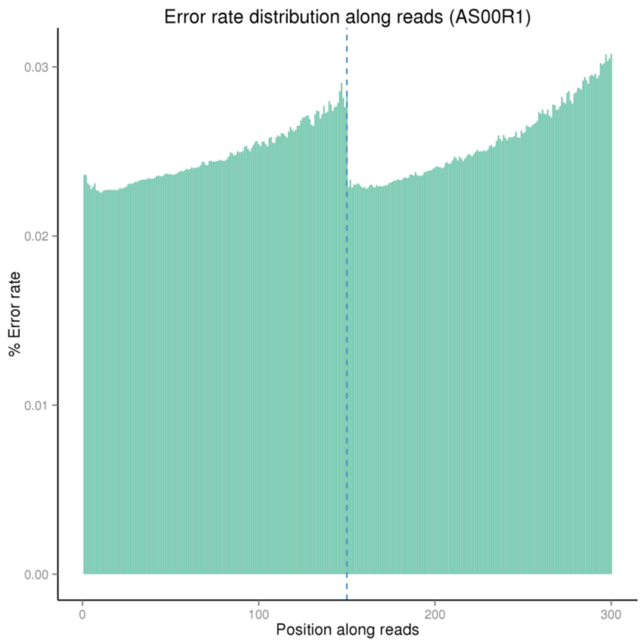

(A) Error Rate

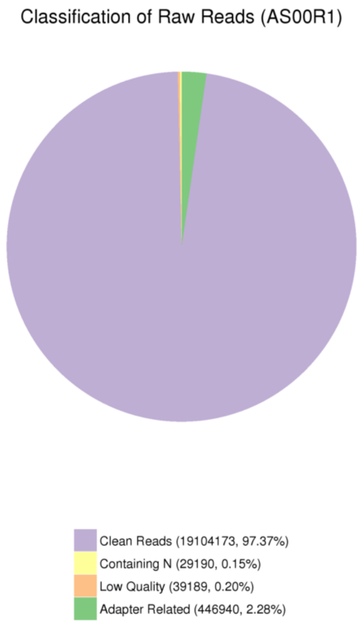

(B) Composition of Raw Reads

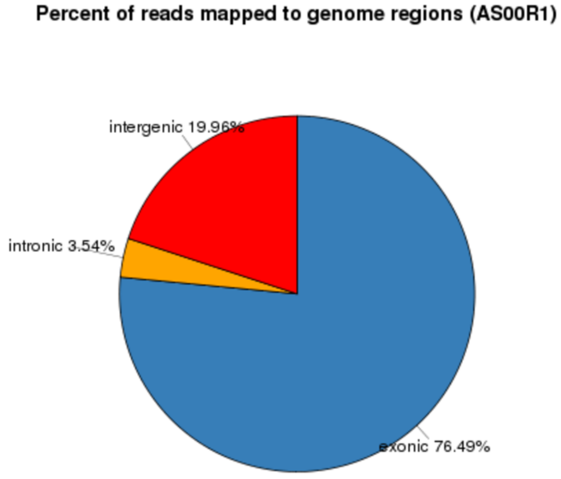

(C) Reads distribution to the reference genome

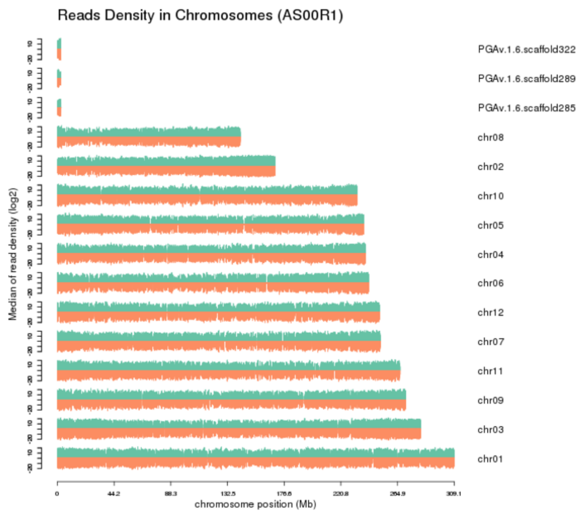

(D) Reads densities in chromosomes

FIGURE 2. Examples for the library obtained from sample 'AS00R1' (Replicate 1 of the time 0 DAA from accession AS).

darker color, and replicates of libraries are set adjacent in both axis, while samples are ordered by accession at each axis. The higher correlation ($r = 1$; darkest color) is obviously present between each library with itself, which is shown in the main diagonal of the matrix. In Figure 6d samples are ordered at each axis by genotype (accession) and time (neighboring times are closer), and we can see a pattern of 4 $\times$ 4 'squares' corresponding to each one of the 4 accessions, the squares in the main diagonal correspond to correlations between each accession. In general data were highly consistent; in all cases correlations

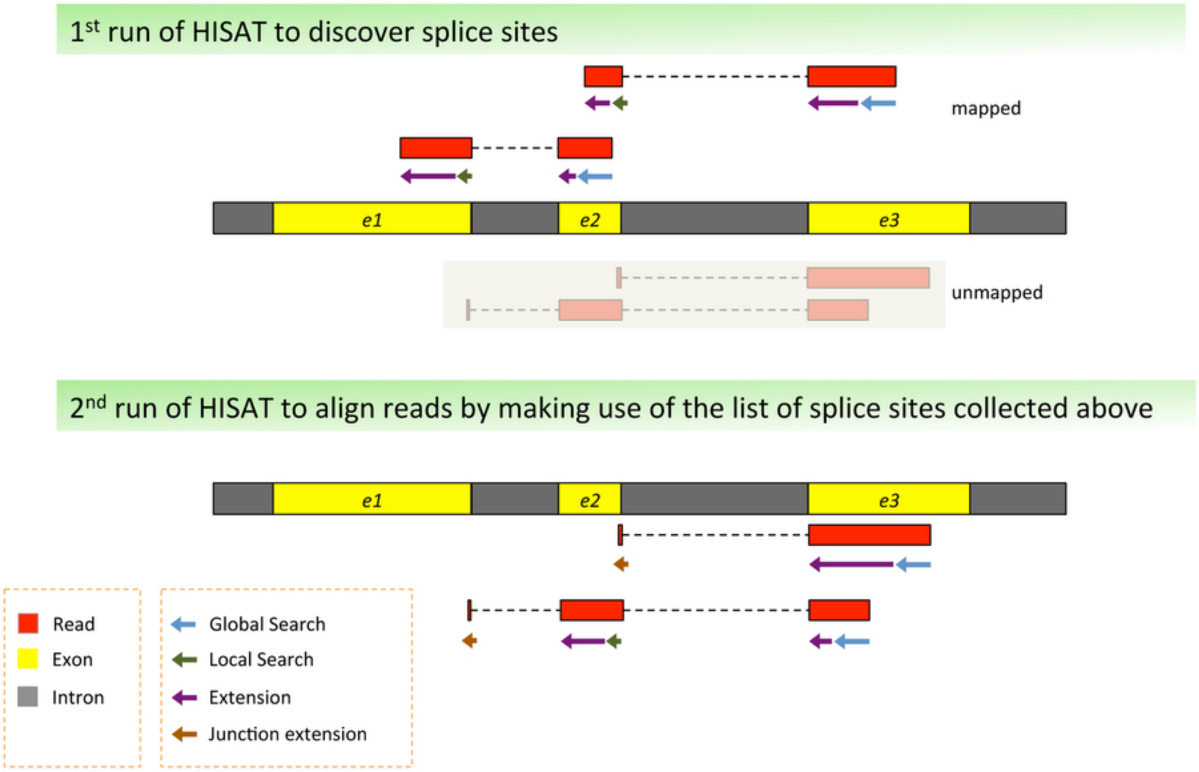

FIGURE 3. Mapping process

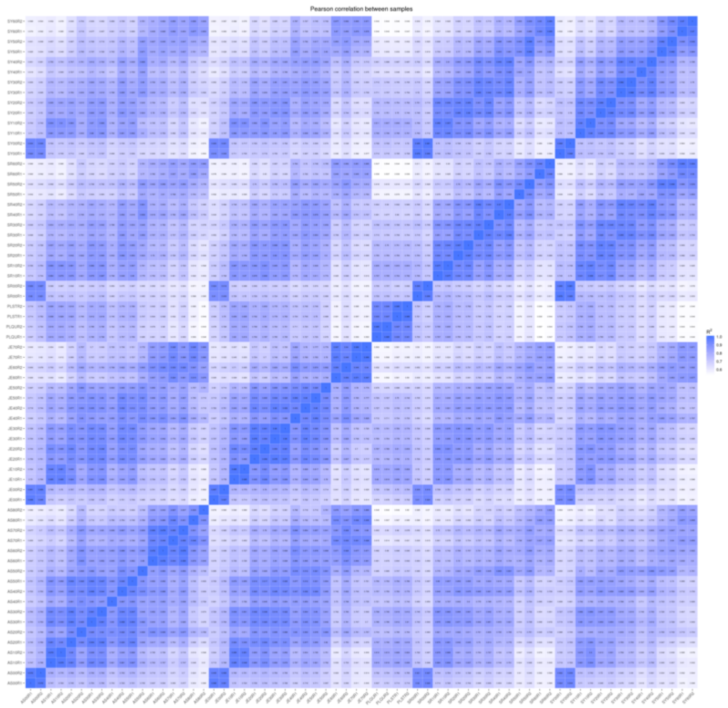

FIGURE 4. Matrix of correlation coefficients between samples (replicates are adjacent).

between replicates of the same accession and time were high and there was gradient from higher to lower correlations depending on time.

Novogene results also included all known Gene Ontology GO and Kyoto Encyclopedia of Genes and Genomes KEGG annotations of the *Capsicum* genome.

All results from Novogene were downloaded and kept in an in-site MySQL relational data base called 'SALSA'.

S-2. Standardized Expression Profile (SEP) estimation

The majority of RNA-Seq studies (Wang et al., 2009) are focus on the direct estimation of differential gene expression. However, in our case we want to estimate the *expression profile*, i.e., the change of the relative gene expression through time. Given that our experiment was an RNA-Seq time-course (Luan and Li, 2003; Iglesias-Martinez et al., 2016) study, the emphasis was to summarize the changes that occur from one point in time to the next. We sampled seven times during fruit development, say $t_1, t_2, \cdots, t_7$, corresponding to 0, 10, 20, 30, 40, 50 and 60 DAA, thus the contrasts of interest were between times t_i, t_{i+1}; $i = 1, 2, \cdots, 6$; i.e., between the six neighboring intervals. Let's denote the true mean gene expression for a given gene within one of the accessions as $\mu_i; i = 1, 2, \cdots, 7$. Then, for each neighbor interval we had three possibilities, say, gene expression decreases from time i to time $i+1$, denoted as 'D' and expressed by the hypothesis $\mu_i > \mu_{i+1}$; steady gene expression from time i to time $i+1$, denoted by 'S' and corresponding to $\mu_i = \mu_{i+1}$ and finally gene expression increases from time i to time $i+1$, denoted as 'I' corresponding to $\mu_i < \mu_{i+1}$. To decide between these alternatives we employed the program edgeR (Robinson et al., 2010) as described below.

It is important to realize that we want to statistically summarize a gene expression profile that exist in the six dimensional space created by the contrasts at neighbor intervals, and thus six tests of hypothesis, one for each one of the neighboring intervals, need to be performed. Given that we test multiple hypotheses (one for each interval), we need to consider the Bonferroni correction (Abdi, 2007) for the probability of calling two expression profiles as statistically different. Thus, to obtain an approximate probability of Error Type I, p^*, when performing 6 tests, we need to use a p^* value equal to $p^* = p^6$, where p is the value employed at each one of the 6 individual tests. In our case we fixed p^* to be equal to 0.01 or 1%. Note that we were not going to directly perform or use hypothesis tests between different gene expression profiles, but only use the expression profile as a reasonable summary of gene expression through time. The basic idea behind this method of estimation was previously published by our group in Martínez-López et al. (2014).

To obtain the p^* values needed by the method, we run edgeR (Robinson et al., 2010) on the matrix of raw counts of reads for each one of the accessions, performing the tests for each gene in contrasts t_i *vs.* t_{i+1}, $i = 1, 2, \cdots, 6$, i.e., for the differences in expression between the 6 pairs of neighboring intervals.

Because at each time interval we had three possibilities for the change of gene expression, as said before, 'D' when $\mu_i > \mu_{i+1}$; 'S' when $\mu_i = \mu_{i+1}$ and 'I' when $\mu_i < \mu_{i+1}$, we call the realization of these profiles 'Ternary Models', because only 3 possibilities were contemplated at each one of the neighboring intervals. Ternary Models can be represented by the six successive results obtained in the intervals; for example, model 'SSSSSS' represent the case where gene expression was steady, i.e., with no significant change during all fruit development, while model 'DDISS' denotes the case where expression decreased from 0 to 10 and 10 to 20 DAA, then increased from 20 to 30 DAA and then stayed steady in the last two intervals, from 40 to 50 and 50 to 60 DAA. Thus, by counting all possibilities we had a total of $3^6 = 729$ different Ternary Models.

To obtain raw estimated expression profiles we calculate, for each gene within each accession, the mean gene expression of the two biological replicates in FPKM units (Mortazavi et al., 2008)[1]. This gave a vector of seven numbers, say $\mathbf{m} = (m_1, m_2, \cdots, m_7)$, corresponding to the seven times points where the expression was estimated. The algorithm to obtain the Ternary Model profile from the raw estimated expression profile, $\mathbf{m}$, needs also the 6-dimensional model vector

$$\mathbf{M} = (M_1, M_2, \cdots, M_6)$$

which contains the letters that denote the change at each one of the 6 intervals; i.e. $M_i \in \{D, S, I\};\ i = 1, 2, \cdots, 6$, i.e., the Ternary Model for the gene.

The algorithm to calculate the Ternary Model profile is presented in the next list.

Algorithm to obtain the Ternary Model profile 'o' from input $\{\mathbf{m}, \mathbf{M}\}$.

(1) Input $\mathbf{m}$ and $\mathbf{M}$; initialize a seven numerical vector, $\mathbf{o} = (o_1, o_2, \cdots, o_7)$ with 'NA' in all its elements and also auxiliar variables $i = 1,\ j = 0,\ k = 0,\ s = m_1$.

(2) (main loop): ***while***$(i < 6)$ {

- ***if***$(M_i = S)$
  $\{s = s + m_{i+1},\ j = j + 1,\ k = k + 1\ \}$
  else
  $\{t = s/j,\ \mathbf{o}[\min(sub) = \text{'NA'}\text{: } (k+1)] = t,\ s = v_{i+1},\ j = 1,\ k = i\}$

(3) $i = i + 1$ } (ends main loop).

(4) # (Examine last element of $\mathbf{M}$ and fill element(s) of "$\mathbf{o}$" as needed).

- ***if***$(M_6 = S)$
  $\{s = s + m_7,\ \mathbf{o}[\min(sub) = \text{'NA'}\text{: } 7] = s/(j+1)\}$
  else
  $\{t = s/j,\ \mathbf{o}[\min(sub) = \text{'NA'}\text{: } 6] = t,\ o_7 = v_7\ \}$

(5) output $\mathbf{o}$.

In the algorithm the elements of the output vector "$\mathbf{o}$", denoted by "$\mathbf{o}[\min(sub) = \text{'NA'}\text{: } x]$", are all elements of the vector that were 'NA' from the smallest subindex (sub) to x. The algorithm to calculate the Ternary Model profile obtains a vector, $\mathbf{o}$, in which the values of steady intervals (intervals with 'S' in the model) are fill with the average of the corresponding values of the elements of $\mathbf{m}$. This is so because when there was not statistical significant changes in one or more intervals, the best estimate of the mean expression is given by the average of the corresponding values of $\mathbf{m}$.

A pair of numerical examples illustrate this algorithm, which converts a raw estimated expression profile, $\mathbf{m}$, into the vector, $\mathbf{o}$, which includes the Ternary Model information, $\mathbf{M}$.

Firstly, consider the case of the gene with id=3 in accession AS; for this gene we have $\mathbf{M}$ = 'SSSSSS' (no interval with a significant change) and the rounded numerical values of the raw estimated expression profile are

$$\mathbf{m} = (0.11, 0.05, 0.00, 0.11, 0, 0.12, 0.08)$$

Because none of the changes in expression between neighboring intervals are significant (model is 'SSSSSS'), all seven values of expression are averaged to obtain each one of the the seven values in $\mathbf{o}$, say

$$\mathbf{o} = (0.07, 0.07, 0.07, 0.07, 0.07, 0.07, 0.07)$$

Secondly, consider a gene with a more interesting Ternary Model, say for example gene with id=526 in accession AS, which has $\mathbf{M}$ = 'DSSISS'. This gene decreases from 0 to 10, stays steady from 10 to 30,

[1] FPKM stands for 'number of Fragments Per Kilobase of transcript sequence per Millions base pairs sequenced'

increments from 30 to 40 and then remains steady up to 60 DAA. The rounded numerical values of the raw estimated expression profile are

$$\mathbf{m} = (39.75, 17.50, 16.61, 18.56, 25.11, 21.20, 16.77)$$

Applying the algorithm to this vector we obtain

$$\mathbf{o} = (39.75, 17.56, 17.56, 17.56, 21.03, 21.03, 21.03)$$

In this case we have $o_1 = m_1 = 39.75$ because in the first interval, M_1 = 'D', we had a significant decrement from the expression at 0 DAA, 39.75, to the expression at 10 DAA, 17.50, but such decrement was followed by two steady states ($\mathbf{M}$ = 'DSSISS'). Now, note that from 10 DAA up to 30 DAA expression was steady, i.e., $M_2 = M_3$ = 'S', thus the values of o_2, o_3 and o_4 are obtained as the average of the values in m_2, m_3 and m_4, i.e., the average of 17.50, 16.61 and 18.56 which equals 17.56, thus $o_2 = o_3 = o_4 = 17.56$. In interval M_4 (from 30 to 40 DAA) we have a significan increment, from $m_4 = 18.56$ to $m_5 = 25.11$, but such increment was followed by two steady intervals, $M_5 = M_6$ = 'S', and thus values of o_5, o_6 and o_7 are equal to the average of 25.11, 21.20 and 16.77 which is 21.03.

Note that vectors of expression profiles, $\mathbf{o}$, are not standardized to have a mean over time of 1 and a standard deviation of 1. Thus the last step to obtain Standardized Expression Profiles (SEPs) is to subtract the mean and divide by the standard deviation all elements of $\mathbf{o}$, say to obtain the SEP, $\mathbf{s}$ from $\mathbf{o}$ we standardize setting $n_i = (o_i - \bar{\mathbf{o}})/S_{\mathbf{o}}$, where $\bar{\mathbf{o}}$ is the average of the seven elements of $\mathbf{o}$ and $S_{\mathbf{o}}$ is the standard deviation of the elements of $\mathbf{o}$.

In the second example (gene with id=526 in accession AS) we have that $\bar{\mathbf{o}} = 22.21$ and $S_{\mathbf{o}} = 7.93$, thus the final representation of the Standardized Expression Profile (SEP), $\mathbf{s}$, is given by

$$\mathbf{s} = (2.21, -0.59, -0.59, -0.59, -0.15, -0.15, -0.15)$$

which has an average of 0 and a standard deviation of 1, i.e. it is 'standardized'.

In summary, the estimation of a SEP, $\mathbf{s}$, proceeds following the steps $\{\mathbf{M}, \mathbf{m}\} \Rightarrow \mathbf{o} \Rightarrow \mathbf{s}$, and it takes into consideration the mean gene expression $\mathbf{m}$, which for each time is the average resulting from two RNA-Seq libraries as well as the statistical significance between neighboring times, contained in the Ternary Model $\mathbf{M}$, adjusting the expression at each time to reflect significant changes by averaging the expression intervals where there is not significance, obtaining the Ternary Model profile, $\mathbf{o}$, to finally obtain the SEP, $\mathbf{s}$, by standardizing $\mathbf{o}$. Even when this procedure could be judged as highly convoluted, it has a great advantage: It allows to compare gene expression profiles throughout time independently of the raw gene expression and it integrates the available statistical evidence for expression change between neighboring times.

Figure 5 shows the plot of the SEP for gene with id=526 in accession AS, presented before as second example above. Additionally to showing the best estimates of standardized changes in expression throughout time, we can see how the SEP model preserves the relative magnitude of expression changes; by observing this plot we can immediately notice that the change from 0 to 10 DAA, with a total absolute difference of 2.8 standardized units, is much larger than the change from 30 to 40 DAA, which has a total absolute difference of 0.74 standardized units.

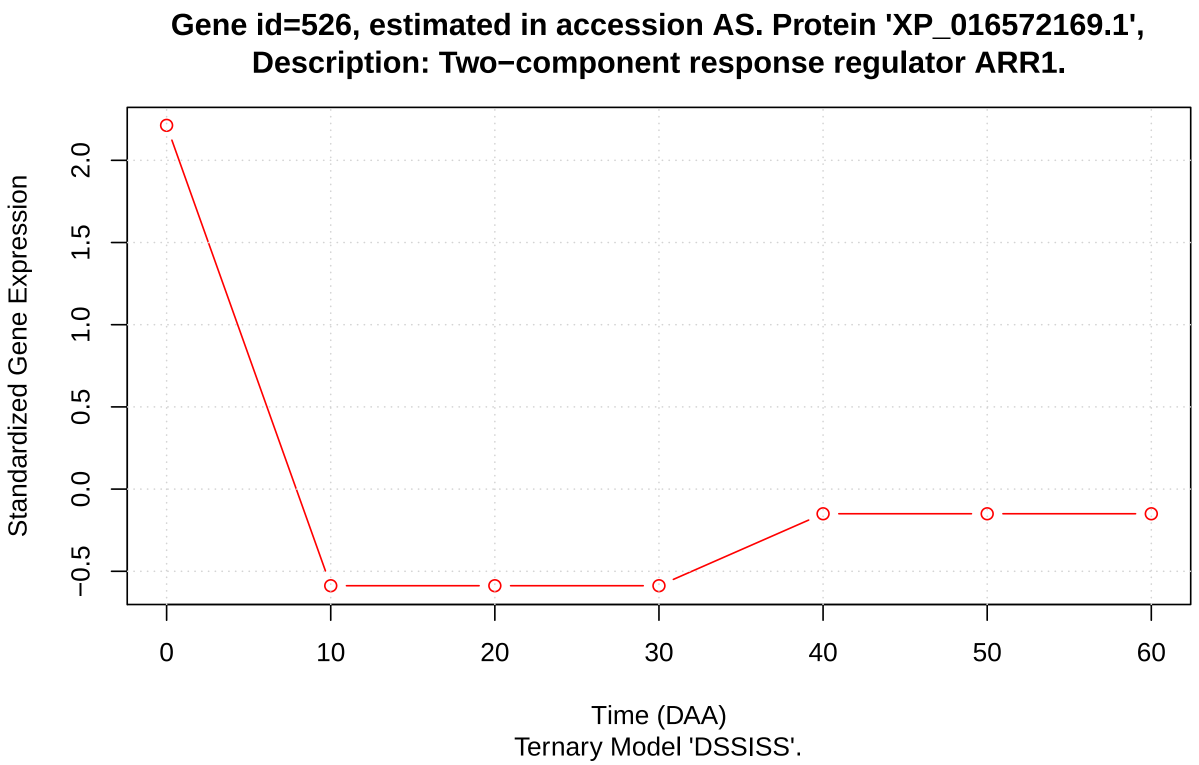

FIGURE 5. Example: Standardized Expression Profile (SEP; **s** vector) for gene with id=526 in accession AS.

S-3. Analysis of late maturing times

As seen in column **Maturity (DAA)** of Table 1 in main text, three D accessions, AS, CW and JE, have times to reach the fully ripe fruit state larger than 60 DAA; 70 DAA for CW and JE and 80 DAA for AS. In this section we make an in depth analysis of gene tendencies both, in the time period from 0 to 60 DAA sampled in the 6 D accessions, as well as in the late maturation times of accessions AS, CW and JE.

S-3.1. **Gene tendencies during fruit development (from 0 to 60 DAA).** Given the SEP methodology, each one of the the genes at each one of the accessions and time intervals is classified into three categories: "D" (Decreasing), when the gene presents a significant expression decrement from the initial to the final points of the interval, or "S" (Steady) when the change of expression was not statistically significant or, finally, "I" (Incrementing) when the gene presents a significant expression increment from the initial to the final time point in the corresponding time interval. Figure 6 presents bar plots for the percentages of the classifications of SEPs into these three tendencies (D, S, I) for all genes in all accessions and time intervals (panel "A"), all genes at all times classified per accession (panel "B") or all genes in all accessions classified per time (panel "C"), while panel "D" presents the plot of percentages of increasing (I), active states (D+I) as well as decreasing (D) and increasing (I) proportions per time interval. The total number of different SEPs analyzed in D accessions is equal to 134,562, corresponding to 22,427 different genes times 6 different accessions. However we have 6 different time intervals, thus the total number of cases classified in Figure 6 is $22427 \times 6 \times 6 = 807,372$.

Percentages of "D", "S" and "I" categories reflect relative transcriptomic activity; "D" and "I" mean significant changes while the "S" state means relative inactivity. In panel "A" of Figure 6 we see that approximately 50% of all cases (all genes in all accessions and time intervals) are in the steady state ("S"), while more than 25% (dashed black line) presented a decrease ("D"), and less than 25% presented an increase ("I").

In panel "B" in Figure 6, which presents percentages of categories for all genes and times classified per accession, we can see a relative heterogeneity in the proportions of genes in steady state ("S"); ordering accessions by the percentage of "S" (in decreasing order) we find CW, AS, ZU, JE, ST and CM, with approximated percentages of "S" equal to 58, 55, 49, 49, 48, and 43 respectively. Thus, accession CW was the one with more steady genes during fruit development (58%), while CM was the one with less steady genes during fruit development (43%), and the difference between those extreme accessions, 58?43 = 15%, is large, showing that different genotypes present different proportions of relatively steady genes during fruit development. On the other hand, in all 6 accessions the proportions of decreasing genes ("D") is larger than the proportions of the ones with an increasing tendency ("I").

Panel "C" in Figure 6 presents percentages of categories for all genes and accessions classified per time interval. This plot shows an heterogeneity in the proportions of genes in steady state ("S") even larger than the one observed when the grouping was performed by accession (panel "B" in Figure 6), and this in turn means that the factor "time of development" has larger effects in the transcriptomes than the ones produced by the genotype (accessions). Ordering time intervals by the percentage of genes in the steady state, "S", in decreasing order, we find that the approximate percentages are 64, 59, 54, 50, 49 and 25 for time intervals "20 to 30", "30 to 40", "40 to 50", "50 to 60", "10 to 20" and "0 to 10", respectively. This can be better appreciated in the panel "D" of Figure 6, which plots the general tendency for all genes in all accessions at the six time intervals. In this plot the grey line (as the grey bars in panel "C") presents the percentage of SEPs in steady ("S") state, while the dark violet line present the sum of the percentages of decreasing ("D") and increasing ("I") cases, i.e., the proportion of active genes, which changed their expression in the corresponding interval. From this panel we can notice the progression of the percentage of active genes throughout chili fruit development. The rounded percentages are 75, 51, 36, 41, 46 and 50%, respectively for the 6 consecutive time intervals. The maximum proportion of active gene expression changes happens at the first interval, from the mature flower at 0 DAA to the 10 days old

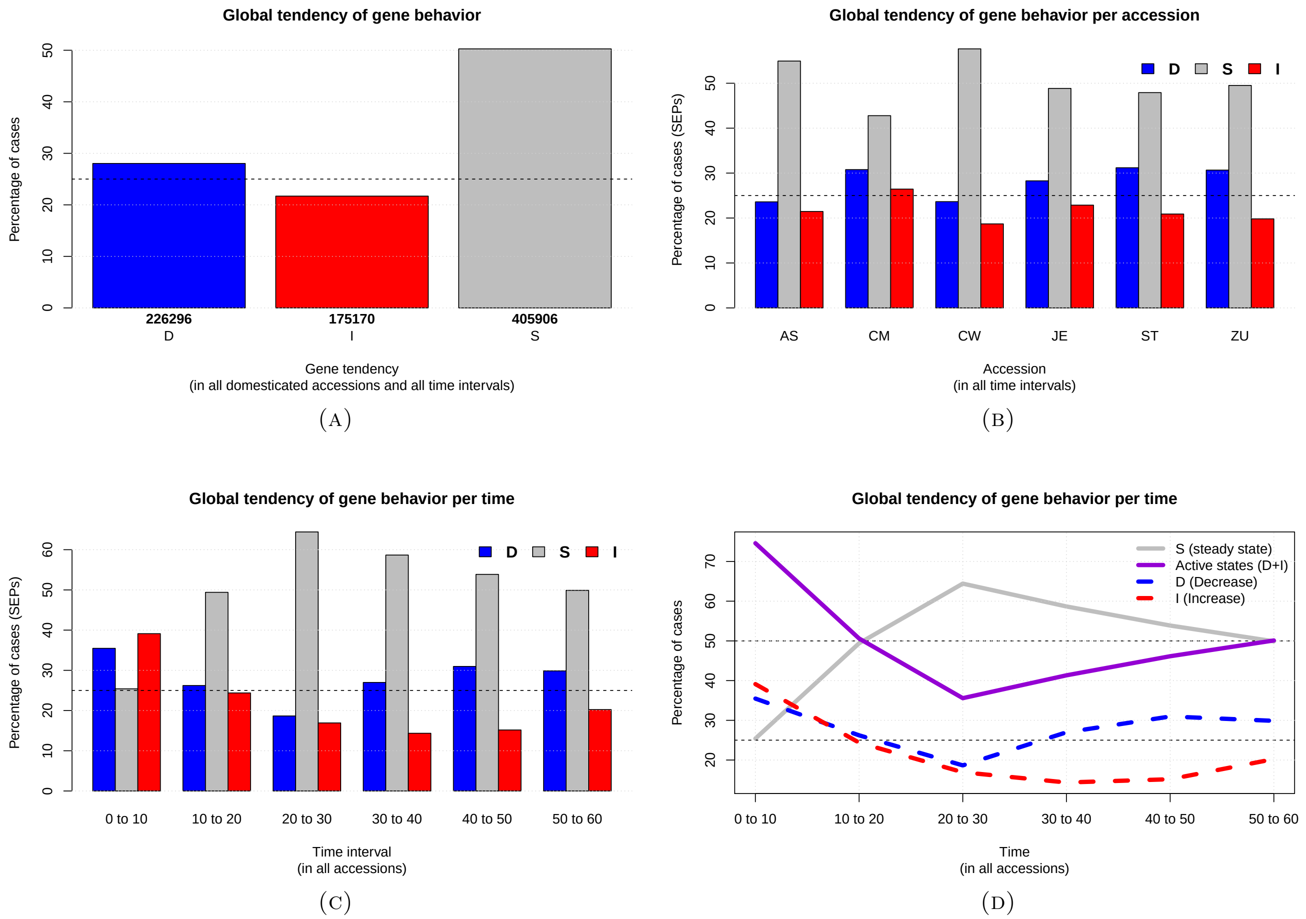

FIGURE 6. Gene Tendencies (D - Decrement, S - Steady and I - Increment) for the 22,427 genes in all D accessions and times (panel "A") as well as subdivided by accession (panel "B") and time intervals (panels "C" and "D").

fruit, and from that interval there is an almost linear decrease in the proportion of active genes, which reach its global minimum at the 20 to 30 DAA interval, where in most accessions the fruit is reaching its maximum size.

From the minimum point in the proportion of active genes at interval 20 to 30 DAA, such proportion increases linearly up to the end of of the time points sampled, the interval from 50 to 60 DAA (panel "D" in Figure 6). Summarizing from panel "D" in Figure 6; maximum of transcriptome activity ($\approx$ 75%) happens at the mature flower (0 DAA). From that point and up to 30 DAA transcriptome activity decreases to reach its minimum, and on that time interval the proportion of genes decreasing (dashed blue line) as well as the proportion of the ones increasing (dashed read line) are of similar sizes. From 30 DAA up to the end of the sampling period (60 DAA), the proportion of transcriptome activity increases linearly (violet line), but on that period the proportions of genes decreasing (dashed blue line) and increasing (dashed read line) are asymmetric, i.e., the proportion of genes decreasing its activity increases, while the one for genes increasing stays low at less than 25%.

S-3.2. **Gene tendencies in late accessions (AS, CW and JE).** As seen in Table 1 of the main text, three of the accessions, AS, CW and JE, have a FRS $>$ 60 DAA; for CW and JE full maturity is reached at 70 DAA, while for JE this stage is reached at 80 DAA. To complete the analysis of gene tendency in these late accessions, we performed the following contrasts between neighboring time intervals: "60 *vs.*

70 DAA" for AS, CW and JE and "70 *vs.* 80" DAA for AS in the 22,374 genes expressed in all RNA-Seq libraries. Contrasts were performed by the edgeR software (Robinson et al., 2010) and results were filtered to get a 1% of False Discovery Rate (FDR) (Benjamini and Hochberg, 1995). Table 1 presents the numbers and percentages of significant tests in these contrasts.

TABLE 1. Numbers and percentages of significant tests in 4 contrasts in late accessions.

	n Significant tests:				n_S	Total
	0	1	2	3		
Number of genes	21,741	505	124	4	633	22,374
Percentages	97.17	2.26	0.55	0.02	2.83	100

In Table 1 we can see that only a small proportion of the genes studied, 2.83%, corresponding to 633 genes, was significant in at least one contrast; only 2.26, 0.55 and 0.02% of the genes were significant (FDR=1%) at 1, 2 and 3 of the contrasts, respectively, and no gene was significant at all the four contrasts.

To reach an approximate 1% FDR between SEPs estimated in time expressions from 0 to 60 DAA a less stringent criterion per interval was employed (Martínez et al., 2020). Figure 7 presents gene tendencies for each one of the four contrasts, employing a FDR $= 0.01^{(1/6)}$, and this figure can be fairly compared with panels "C" and "D" in Figure 6.

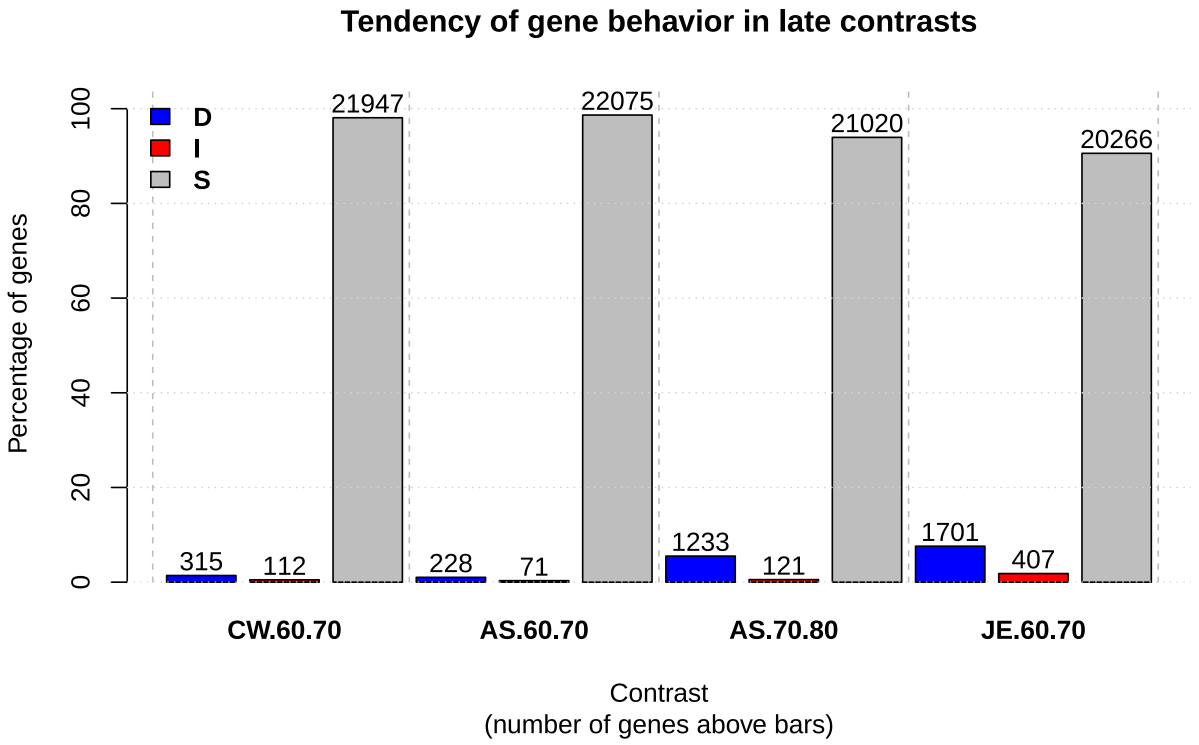

FIGURE 7. Tendency of gene behavior in late contrasts. Contrasts coded by accession key and times contrasted.

In Figure 7 we can see that only small numbers of genes are in an active state ("D" or "I") at each one of the 4 contrasts. The percentages of active genes ("D" + "I") per contrast are 0.95, 0.67, 3.03 and 4.71% for contrasts CW.60.70, AS.60.70, AS.70.80 and JE.60.70, respectively and in all four contrasts the proportion of genes decreasing (blue bars) are larger than the corresponding proportion of genes increasing (red bars); in fact, the ratios of the numbers of "D" over "I" genes are approximately 2.81, 3.21, 10.19 and 4.18 for contrasts CW.60.70, AS.60.70, AS.70.80 and JE.60.70, respectively.

In summary, transcriptome changes above 60 DAA in the accessions with a larger fruit development time (AS, CW and JE), involve a small number of 633 differentially expressed genes which can be analyzed independently of the full set of 22,374 genes for which we have SEPs which include fruit development between 0 and 60 DAA.

S-3.3. **SEP differences between 'normal' and 'late' accessions.** Within the time period included in the SEPs, i.e., 0 to 60 DAA, we will investigate if there are significant differences in expression time profiles between accessions with 'normal' maturation times ("CM", "ST", "ZU") and those with late maturation times ("AS", "CW", "JE"). We used "*Salsa*" function "`analyze.2.SEPs()`", which test Euclidean distances between and within SEPs, see Escoto-Sandoval et al. (2020), to decide if the time profiles present a significant difference. Of the 22,374 test performed, only 37 of them, less than 0.17% of the total of 22,374 genes studied, resulted significant with a FDR of 1%.

Figure 8 presents SEPs plots for the gene coding for protein XP_016577952.1, the chromoplastic *capsanthin/capsorubin synthase*, which is expressed only in chromoplasts at late maturing states in the *Capsicum* fruit (Kothari et al., 2010; Gómez-García and Ochoa-Alejo, 2013; Martínez-López et al., 2014; Tian et al., 2015). In this figure thick colored lines in red and blue show the average of SEPs at each expression time, and thin vertical lines are 95% confidence intervals (CIs) for the corresponding means, while thin pale lines in pink and grey show the behavior of individual accessions. In this plot we can see that the mean expression of the gene stays steady at negative standardized expression from 0 up to 40 DAA, a point where id begins to increase in expression up to 60 DAA. This behavior is not significantly different in normal and late maturing accessions ($P = 0.24$; $Q = 0.83$).

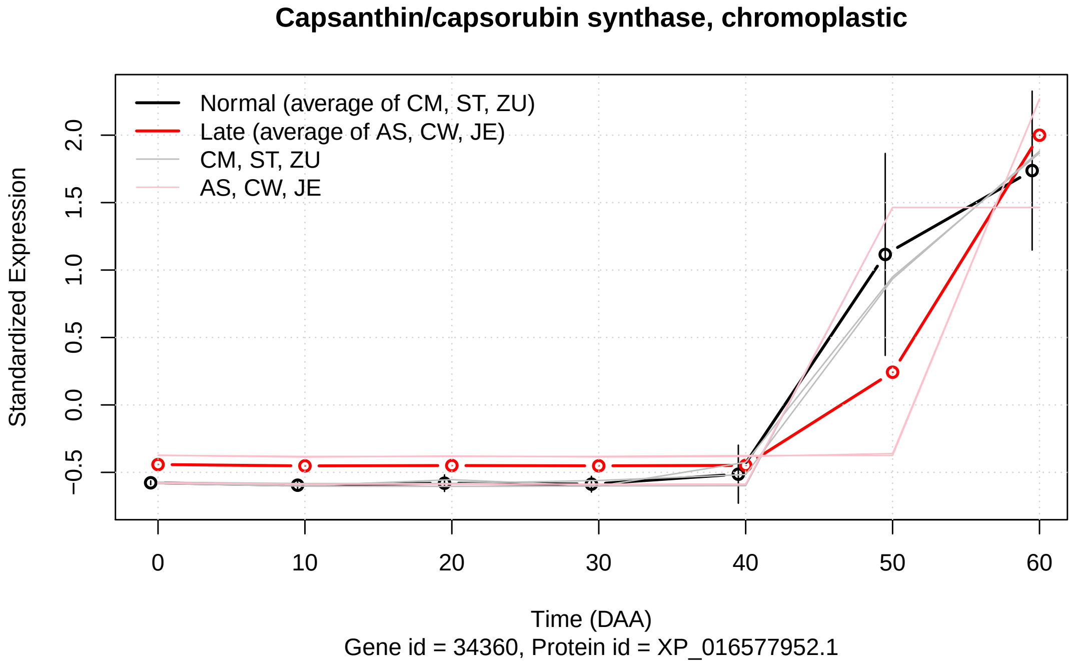

FIGURE 8. Example of SEPs in a gene that does not present differences between normal and late maturity accessions. P-value = 0.24, Q-value = 0.83

Given that only 37 genes present significant differences between SEPs in accessions with normal and late maturation times, we concluded that using times between 0 and 60 DAA was reasonable *via* SEPs will not induce a bias in the results.

S-4. Testing differences between Domesticated (D) and Wild (W) SEPs

The focus of this work was the detection of changes in standardized expression profiles (SEPs) between D and W accessions caused by the domestication process. We studied 10 accessions, 6 D and 4 W (see Table 1 in the main text), and found that a total of 22,427, representing approximately 64% of the genes annotated in the *Capsicum* genome (CM334 v1.6) were consistently expressed in all 10 accessions at one or more of the times sampled and in more than one of the two biological replicates per accession.

For each gene we had 10 SEPs, 6 from D and 4 from W accessions, and we want to discriminate with a univariate statistic if there were differences in SEPs when grouping them in the D and W sets. For this we selected the Euclidean distance between SEPs, defined as

$$d_{a,b} = d(\mathbf{s}^a, \mathbf{s}^b) = \sqrt{\sum_{i=1}^{i=7} (s_i^a - s_i^b)^2}$$

where $\mathbf{s}^a$, $\mathbf{s}^b$ are two different profiles for the same gene. For a given gene we calculated the total of $10(10-1)/2 = 45$ different distances, $d_{a,b}$; $a \neq b$, and classified those distances into two groups, *distances between* D and W accessions and *distances within* one of the groups. The number of distances between D and W is equal to $6 \times 4 = 24$, while the remaining $45 - 24 = 21$ distances happen within the two groups, say $6(6-1)/2 = 15$ within D accessions and $4(4-1)/2 = 6$ within W accessions.

For a single gene, our interest was to detect significant differences in SEPs between the D and W accessions, and this can be translated to the statistical hypothesis $\mathcal{H}_0 : \mu_b = \mu_w$ *versus* $\mathcal{H}_a : \mu_b > \mu_w$, where μ_b and μ_w are the true means of the distances between and within the D and W groups, respectively. If we accept the null hypothesis $\mathcal{H}_0$ as true, then we have no evidence of differences between SEPs in the D and W accessions, while if this hypothesis is rejected in favor of $\mathcal{H}_a : \mu_b > \mu_w$ (note that this alternative implies a one-tail test), we conclude that the mean distance between the two groups is significantly larger than the distance within those groups, and this implies a difference in SEPs between D and W. To perform the statistical test we assayed a randomization test comparing it with the usual parametric one tail t-test, and found that those alternatives were almost equivalent, opting for the second given the high computational cost of the second and the large number of tests (22427) that needed to be performed.

Figure 9 presents the histogram of the P-values obtained in the 22427 test of the null hypothesis $\mathcal{H}_0 : \mu_b = \mu_w$ *versus* $\mathcal{H}_a : \mu_b > \mu_w$.

An interesting feature in Figure 9 is that the first bar, including P values between between 0 and 0.05, includes 4465 cases, approximately 20% of the total. This indicates that the P distribution of the tests performed is far from being uniform, as expected from randomized tests (Bland, 2013). And because we tested all genes expressed during fruit development, the non-uniformity of the P distribution for the tests implies that selection had an important role in the modification of SEPs.

Table 2 presents the matrix of average mean distances between 22427 SEPs, corresponding to equal number of genes, in the 10 accessions.

In Table 2 we can see that the minimum of the mean distances, 1.63 (in blue), occurs between SR an SY, two W accessions, while the maximum, 2.40 in red, happens between AS and SR as well as between AS and ST, in both cases a D and W accessions respectively. On the other hand, the mean average distance within D and W accessions (21 values from the matrix) is 2.02, while the mean average distance between D and W accessions (24 values from the matrix) is 2.18; i.e., the D and W accessions form two well segregated groups.

The dendrogram presented in the Figure 1 of the main text was obtained by applying the agglomerative Ward's algorithm on the distance matrix shown in Table 2. In that figure W accessions are grouped in a single cluster (left hand side), well separated at a mean Euclidean distance > 2.8 from the one formed

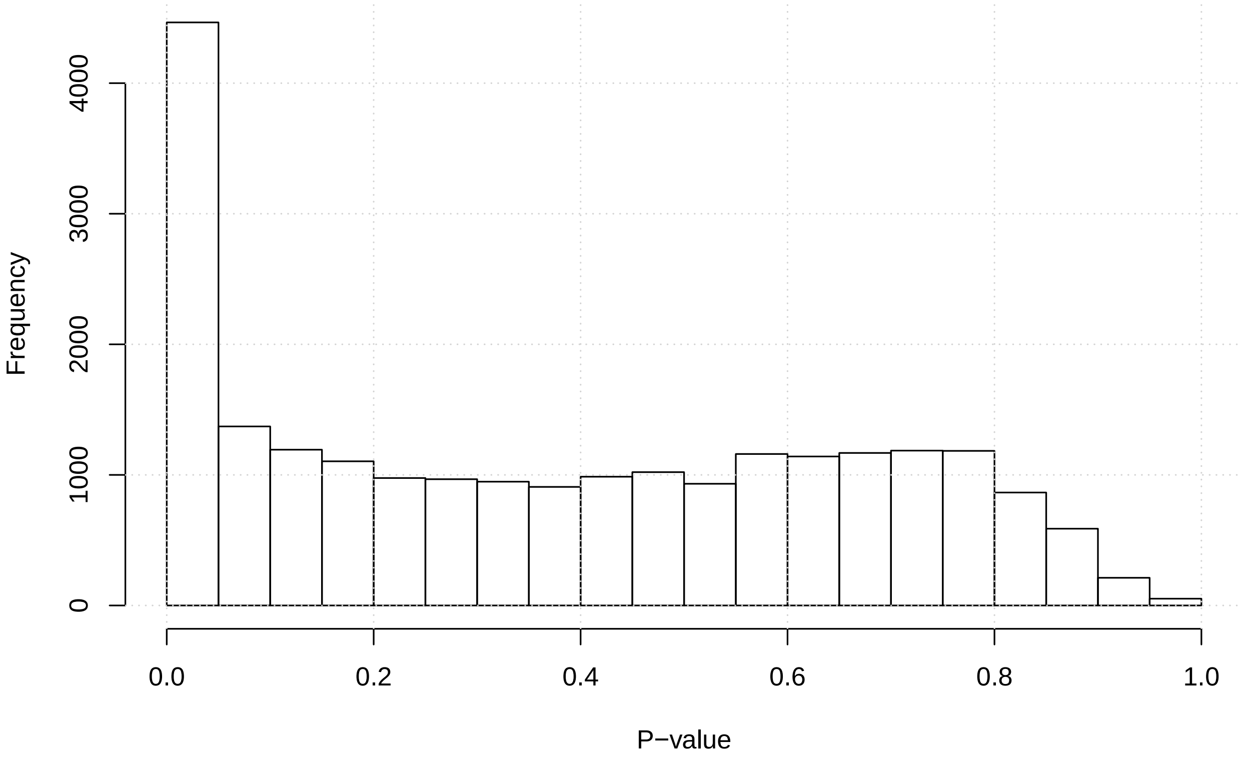

FIGURE 9. Histogram of the P-values obtained in the 22427 test of the null hypothesis $\mathcal{H}_0 : \mu_b = \mu_w$ *versus* $\mathcal{H}_a : \mu_b > \mu_w$ employing the one-tail t-test.

TABLE 2. Matrix of average mean distances between the SEPs in the 10 accessions.

	CM (D)	CO (W)	CW (D)	JE (D)	QU (W)	SR (W)	ST (D)	SY (W)	ZU (D)
AS (D)	2.13	2.33	2.05	1.91	2.35	2.40	2.40	2.37	2.27
CM (D)		1.96	2.07	2.01	1.98	1.95	2.11	1.97	1.98
CO (W)			2.26	2.22	1.80	1.78	2.18	1.74	1.95
CW (D)				2.02	2.29	2.31	2.23	2.31	1.97
JE (D)					2.26	2.29	2.31	2.18	2.08
QU (W)						1.91	2.15	2.00	2.12
SR (W)							2.21	1.63	2.05
ST (D)								2.23	2.06
SY (W)									2.04

by the 6 D accessions (right hand side), this shows that gene expression variability within the W and D groups is smaller than the distance between those groups.

S-5. ANALYSES PER TIME OF SEPS IN D AND W ACCESSIONS

For each one of the 22427 genes expressed during fruit development we have 10 SEPs, and in the previous section we have described the univariate test performed on the Euclidean distances to decide if the SEPs in the set of 6 D accessions could be considered different to the 4 ones in the W group. Independently of the fact that SEPs grouped into the D and W could be considered to be equal or not by that test, we can additionally analyze the differences between SEPs in the 7 stages of development (0, 10, 20, $\cdots$, 60 DAA), grouping a single gene or sets of genes in the D and W sets.

Let's denote as $\mathbf{s}_{\mathbf{n}}^{D}$, $\mathbf{s}_{\mathbf{n}}^{W}$, the 7-dimensional SEP vectors for genes in an arbitrary set of genes $\mathbf{n}$, which cardinality is n, i.e., the set $\mathbf{n}$ is constituted by n different genes ($|\mathbf{n}| = n$).

As an example, define $\mathbf{n}$ as the set formed with the gene with identifier 580 (a single gene). Then $\mathbf{s}_{\mathbf{n}}^{D}$ is constituted by 6 different vectors, each one corresponding to each one of the 6 D accessions, while $\mathbf{s}_{\mathbf{n}}^{W}$ is formed by 4 different vectors, each one corresponding to each one of the 4 W accessions. Now, for each stage of development, $i = 1, 2, \cdots, 7$, we have two sets of independent standardized gene expressions, say, $d_i = \{s_{ij}\};\ j = 1, 2, 3, 4, 5, 6$ for D and $w_i = \{s_{ik}\};\ k = 1, 2, 3, 4$ where the subindex j denote accession, D or W, respectively. Note that all elements $\{s_{ij}\}$, $\{s_{ik}\}$ are fully independent, because each one of them was estimated from a different RNA-Seq library.

For each one of the stages of development, the hypotheses of interest are: $\mathcal{H}_0 : \mu_{\mathbf{n},i}^{D} = \mu_{\mathbf{n},i}^{W}$ *versus* $\mathcal{H}_a : \mu_{\mathbf{n},i}^{D} \neq \mu_{\mathbf{n},i}^{W}$, where $i = 1, 2, \cdots, 7$ and $\mu_{\mathbf{n},i}^{D}, \mu_{\mathbf{n},i}^{W}$ represent the true means of standardized expression at developing stages 0, 10, $\cdots$, 60 DAA, respectively. The number of standardized observations in the sets D and W depend on the number of genes in the set $\mathbf{n}$, as before $|\mathbf{n}| = n$, thus if $n = 1$ (a single gene tested), then the number of observations to be included in the two sets to be tested are 6 for D and 4 for W, while in general for any any set of genes $\mathbf{n}$ with n genes we will have $6n$ and $4n$ observations for D and W, respectively. To perform the tests $\mu_{\mathbf{n},i}^{D} = \mu_{\mathbf{n},i}^{W};\ i = 1, 2, \cdots, 7$ as well as to obtain 95% Confidence Intervals (CIs) for the means of each group at each one of the times we employed the two tail t-test. The procedure to perform the test and plot the results for any arbitrary set of genes was programed in an R function.

On the other hand, it was considered important to evaluate the stage at which the maximum expression of a gene was reached. In this case for each SEP we determine stage (0, 10, $\cdots$, 60) at which the maximum standardized expression is reach. Denote as m_i the point of development at which the maximum of the SEP vector $\mathbf{s}_i = (s_{i1}, s_{i2}, \cdots, s_{i7})$ is found. For example, if $\max(\mathbf{s}_i) = s_{i3}$, this means that the maximum standardized expression took place at the third stage ($i = 3$), corresponding to 20 DAA, thus the value of m_{i3} is 20, etc. For any gene or set of genes $\mathbf{n}$, we calculated the set of maxima in D and W accessions and tested the hypothesis $\mathcal{H}_0 : \Psi_{\mathbf{n},i}^{D} = \Psi_{\mathbf{n},i}^{W}$ *versus* $\mathcal{H}_a : \Psi_{\mathbf{n},i}^{D} \neq \Psi_{\mathbf{n},i}^{W}$, where $\Psi_{\mathbf{n},i}^{D}, \Psi_{\mathbf{n},i}^{W}$ represent the true means of the maximum standardized expression and calculated the corresponding 95% CI.

The functions to analyze and plot the results for an arbitrary set of genes, $\mathbf{n}$, where employed to obtain figures 2, 3 and 4 presented in the main text. In these, as in any results from such functions, the corresponding plots show the 95% CI for mean standardized expression as thin lines at each stage of development, while the estimated mean maximum expression is shown by asterisks with their corresponding 95% CIs shown by an horizontal line. To illustrate these kinds of results we present examples for two genes.

Our first example corresponds to the results obtained for the gene with id=580, and plots are presented in figures 10 and 11.

Figure 10 presents SEPs for the *Capsicum* fibrillin (FBN). Fibrillins are nuclear-encoded, plastid proteins associated with chromoplast fibrils and chloroplast plastoglobules (Singh and McNellis, 2011), and in Figure 10 we can appreciate how expression of FBN is highly concordant in all accessions. In that figure the points plotted are slightly displaced in the X axis (DAA) to avoid line and symbols overlapping. In all accessions TMs for FBN had a low standardized expression from 0 up to 40 DAA, where the expression increases rapidly, reaching the maxima at 50 (in 3 accessions; 2 D and 1 W) or 60 (7 accessions; 5 D, 3 W) DAA. The FBN gene does not present a significant difference in distances between D and W accessions, having a P-value of 0.8 in that test, and exemplifying a case of a gene which was not affected by domestication. On the other hand, Figure 10 presents mean SEPs for the FBN gene. That figure was produced with our function '`TMmean.plot()`', which also produced the output presented in Appendix S-12.

In Appendix S-12 we see that the results include tables of means and CI for the means for the standardized expression at each point in time; those CI are plot as thin vertical lines in Figure 11, allowing the visual

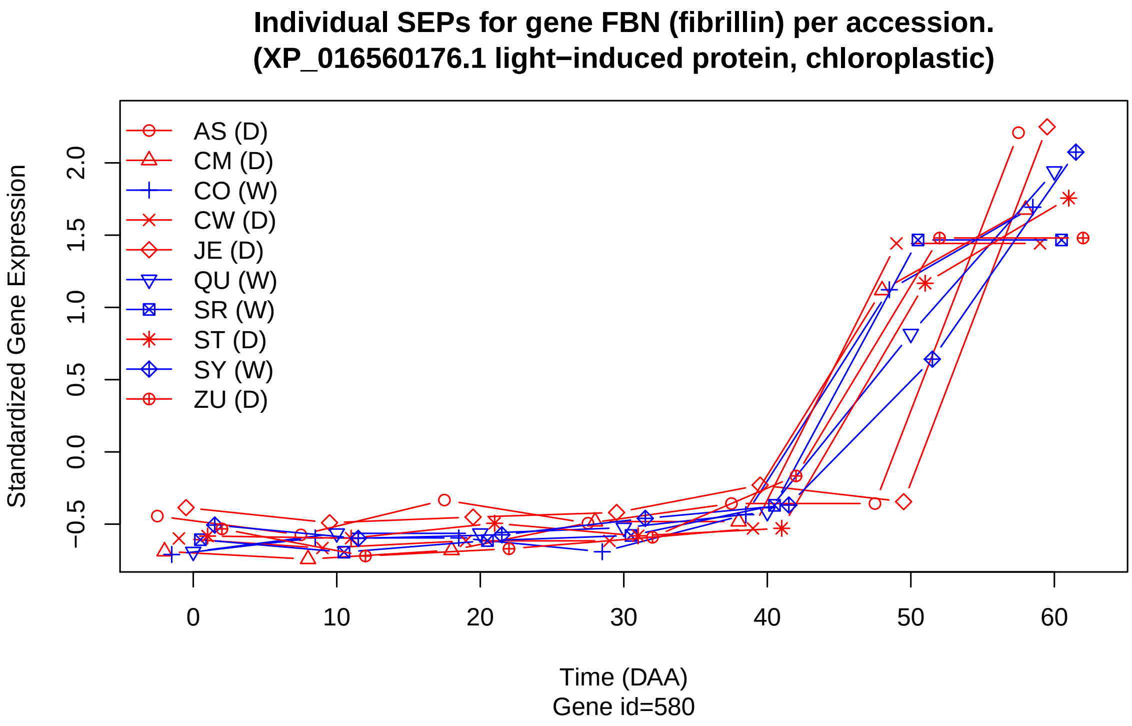

FIGURE 10. SEPs per accession for a gene with highly concordant expression patterns in all 10 accessions. Values per accession were slightly displaced in the Y axis to avoid overlapping.

judgment of the difference between the means in the D (red) and W (blue) sets. For all time points (0, 10, $\cdots$, 60 DAA) we see that the CI of D and W overlap, and the lack of a significant difference can be observed in the P-values for the t-test of means D vs W per time point in Appendix S-12. The second analysis performed is the estimation of means and t-test for the maxima in the D and W groups. Appendix S-12 presents the means and 95% CI for those estimates. The mean for the D group is 56.67 DAA while the mean for the W set is only slightly different, 57.5, with CIs overlap between the two groups. Finally, the lack of significance of the difference in the mean maxima between the two groups is confirmed by the t-test, which gives a value of $P = 0.8065$ The function even gives the interpretation of the result in the line: '(Genes are Early in D but the difference is NOT significant at 0.05)'. Figure 11 presents the means of the times where the maximum expression for each set is estimated as asterisks and the corresponding CI as broad horizontal lines. From all the analyses we can conclude that the FBN gene has a highly similar expression pattern in both, D and W accessions. This kind of analysis and plots were used for figures 2, 3 and 4 in the main text with different sets of genes.

Figures 12 and 13 present plots for a gene with highly different SEPs between D and W and Appendix S-12.1 presents the statistical analysis for this case.

The gene with id=19147, a transcription factor identified as 'B3 domain-containing protein At5g42700-like' and with protein identifier XP_016568750.1, was highly significant ($P < 4.6 \times 10^{-14}$) in the univariate test for differences in SEPs between D and W, and in fact Figure 12 shows that this gene has SEPs which in D accessions have a maximum at 10 DAA, while in W the maximum is present at 30 DAA. This expression pattern indicates that this gene belongs to the group of 'D10W30' genes defined in the main text. Indeed, in Figure 13, which presents the mean SEPs for the gene and the 95% CIs for time of maximum expression over the the X axis, and standardized gene expression over the Y axis, shows that the maxima are different for D and W, while there are significant differences in mean expression at 10,

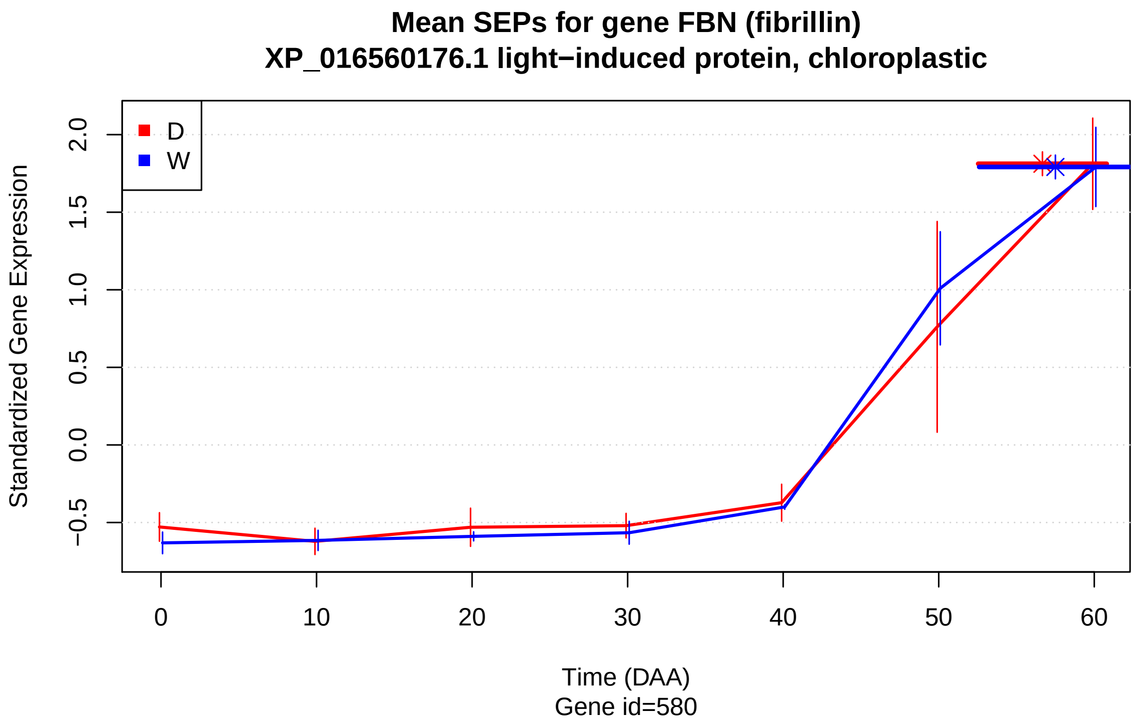

Figure 11. Main lines link the mean SEPs and the thin vertical lines give the 95% CI for the respective estimated points. Asterisks point to the estimated time in DAA where the maximum mean expression was estimated while broad lines over the asterisks are the 95% CI for those points.

30, 40, 50 and 60 DAA. Appendix S-12.1 presents the R output with the statistical results obtained in the analyses.

The same plots and statistical analyses presented in figures 11 and 13 and appendices S-12 and S-12.1 for individual genes can be performed for groups of genes, as done to plot figures 2, 3 and 4 in the main text. To perform statistical analyses of a gene, or sets of genes, we considered contrasts between two groups of accessions, 6 D (AS, CW, JE, ST and ZU in Table 1) and 4 W (CO, QU, SR and SY in Table 1 in main text). In all cases, the null hypothesis was that at each time point the mean expression of the D and W groups was equal, whereas the alternative was that these parameters differed. Variation within the D and W groups was considered as a statistical error (unexplained variation) and a t-test was used to obtain Confidence Intervals (CI) for the means and to evaluate significance at each of the 7 time points sampled. We determined the mean SEPs for different gene groups in the D and W accessions (Figure 14).

The mean for the D and W groups differed significantly (Figure 14 A). At the mature flower state (0 DAA), the standardized mean expression for D was much higher than for W, implying that the average transcription activity in this state is substantially larger for the D genotypes. In the interval between 0 and 10 DAA, the mean standardized expression increased for both groups, although the rate of increase was higher for D. At 10 DAA, the mean expression for D reached a peak value, but for W the increase continued, although at a slower rate, to peak at 20 DAA. From the peak at 10 DAA, the mean expression for D decreased, at different rates, and was lower at all subsequent time points. The lowest value was seen at 60 DAA. In contrast, decreases in the mean expression for W began later, occurring from 20 up to 50 DAA, and reached a minimum of -0.27, which is smaller than the minimum for the D group, -0.25, seen at 60 DAA. The more relevant differences between mean expression profiles between D and W were seen during the intervals between 10 and 20 and 50 to 60 DAA, when the trend (i.e., slope of

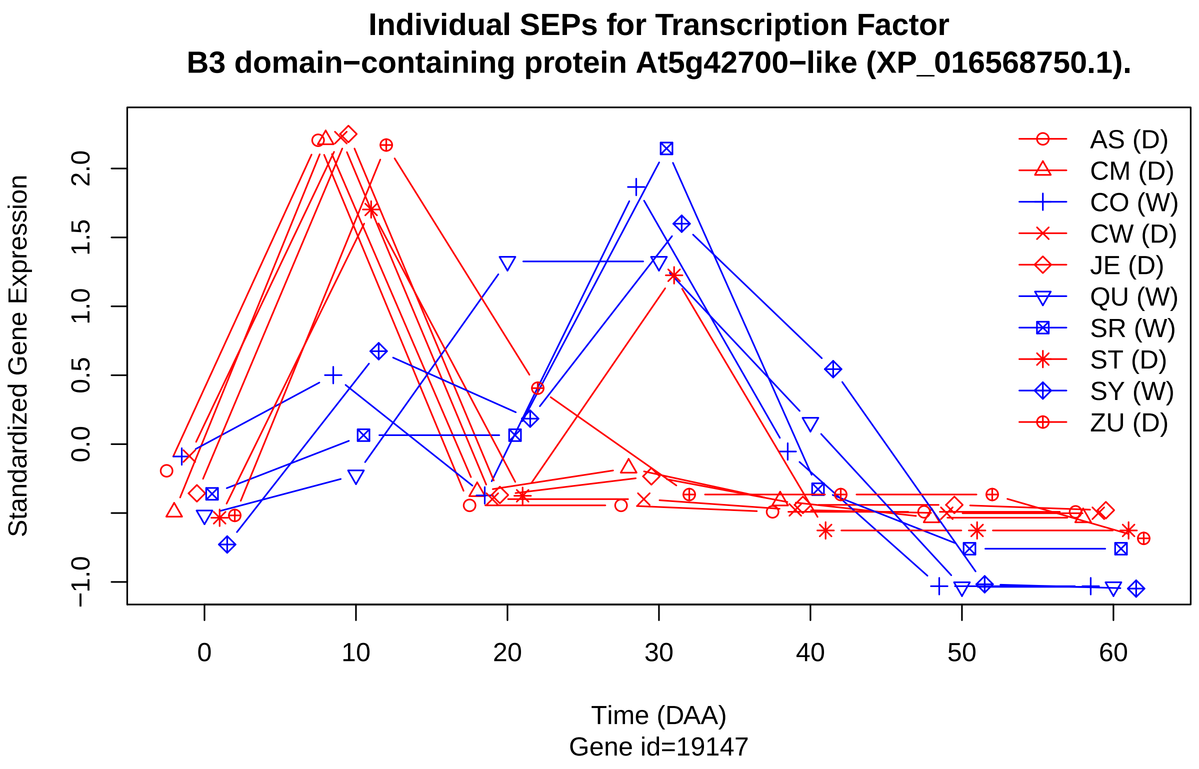

FIGURE 12. SEPs per accession for a gene with highly different expression patterns between D and W. Values in Y axis slightly displaced to avoid overlap.

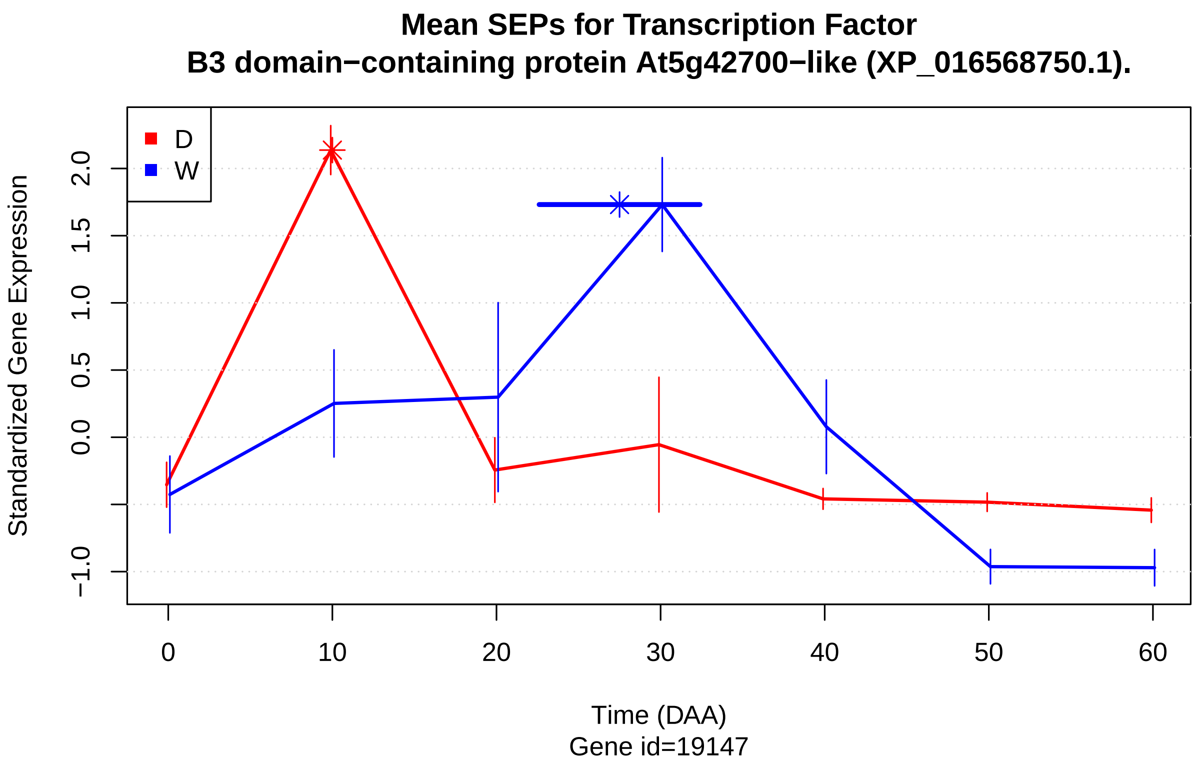

FIGURE 13. Mean SEPs per accession for a gene with highly different expression patterns between D and W.

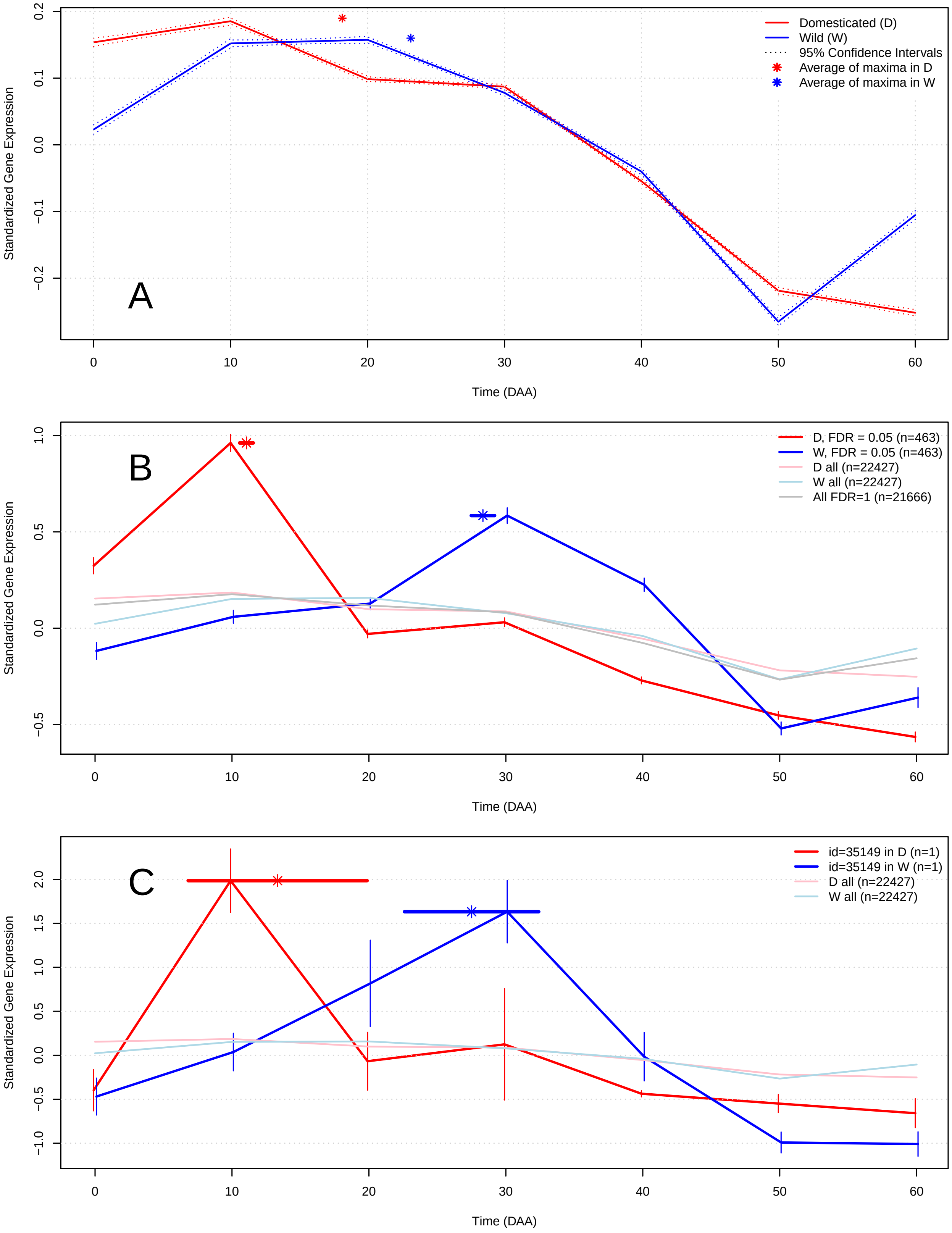

FIGURE 14. **Mean SEP (Standardized Expression Profile) for groups of genes in Domesticated (D) and Wild (W) accessions.** Continuous colored lines link the means of standardized gene expression at each time point. (A) Complete set of expressed genes (n=22,427). (B) Set of genes having differential expression profiles between D and W (n=463; FDR=0.05). Pale colors indicate the expression profile for all genes, and the gray line represents genes that had no difference in expression between D and W (FDR = 1). (C) Expression profiles for the gene (n=1) encoding the protein "G2/mitotic-specific cyclin S13-7" (XP_016543946.1). In B and C the thin vertical lines represent the 95% CI for the means. Asterisks indicate the mean time of maximum expression and the horizontal lines over the asterisks represent the 95% CI for the mean at each time point.

the regression models) was inverted such that D was decreasing while W was increasing. On the other hand, less marked differences between D and W were seen between 30 and 50 DAA when the mean standardized expression decreased nearly in parallel for both groups. The average of the time at which the maximum expression was reached in each group (marked by asterisks) was five days earlier for D than W. All observed differences were significant.

Differences in SEP of individual genes varied between D and W. To select the genes having the largest differences between D and W, we applied a statistical test on individual differences and used a False Discovery Rate (FDR) threshold of 0.05, which for these tests produced a P value < 0.000002. Using these criteria we selected a set of 463 genes, representing approximately 2.06% of the total (Figure 14 B). The expression profiles of these 463 selected genes differed markedly between D and W, ranging from -0.56 (D at 60 DAA) to 0.96 (D at 10 DAA), which is much larger than the range of variation for the means of all genes (Figure 14 B, pale red and blue lines). The profiles for these genes also completely differed from the average profile of genes that had similar expression profiles in both D and W (grey line, FDR = 1). The differences in expression profiles between D and W were well defined and significant; the peak of mean expression for D occurred at 10 DAA, while the peak for W occurred later, at 30 DAA. The average time of maximum expression (asterisks with corresponding 95% CIs) was 11.06 DAA for D and 28.33 DAA for W, or a difference of -17.27 DAA. Of the 463 selected genes, 36 ($36/463 \approx 0.08$; 8%) are transcription factors (TFs). This percentage is higher than that for TFs annotated in the Capsicum genome ($1{,}859/34{,}986 \approx 0.05$ or 5%). A list and description of the 463 selected genes and details of statistical analyses are presented in the Supplemental SG and SM-4, respectively.

We next focused on the expression profiles in the D and W accessions for a single gene encoding the protein 'G2/mitotic-specific cyclin S13-7' (Figure 14 C). For this gene, the 95% confidence intervals (CIs) for the means at each time (thin vertical lines), as well as for the average of the time at which maximum expression was reached for each group (horizontal lines over the asterisks) was longer, since the means were obtained from only one gene (n=1) and thus each point is obtained from only individual data for the 6 and 4 accessions for D and W, respectively (see Methods). Nevertheless, the sample size and statistical method employed show that there are significant differences between the D and W profiles for a single gene, given that the 95% CI values do not overlap (Figure 14 C).

The results indicate that the design and results of this experiment showed differences in expression profiles between D and W at the level of whole gene sets (Figure 14 A), groups of particular genes (Figure 14B), and individual genes (Figure 14 C). Taking these findings together, we can thus conclude that there are relevant differences in expression profiles between domesticated and wild varieties of chili peppers.

S-5.1. **Differences in Expression of Genes Related to Cell Reproduction Appear Earlier and are Larger in Domesticated than Wild Genotypes.** Based on the evidence that mean SEP differ between the D and W accessions, we investigated differences in expression profiles in groups of genes related to particular biological processes. We first examined the mean SEPs of a group of 1,125 genes associated with cell reproduction (Figure 15).

We observed that the mean tendency of all 1,125 genes (solid lines) and a subset of 170 genes showed significant ($P < 0.01$) differences in expression profiles between D and W (dashed lines; Figure 15 A). Moreover, significant differences between D and W were observed at all 7 time points for both the entire group and gene subset. For both groups (n=1,125 and n=170), the mean expression was higher in D than for W at 0, 10 and 50 DAA. Meanwhile, the intervals from 10 to 20 and 50 to 60 DAA had contrasting tendencies for D and W. For both intervals the mean expression decreased for D, but increased for W. The peak of mean expression occurred earlier for D (at 10 DAA) than for W (at 30 DAA) and the magnitude of expression at the peak was also much larger for D than for W.

The mean expression value for 235 genes that are directly annotated in the cell cycle —but not in other cell reproduction processes— was significantly higher and occurred earlier for D compared to W, as evidenced by the peak of 0.3 standardized units at 10 DAA for D and 0.2 standardized units 30 DAA for

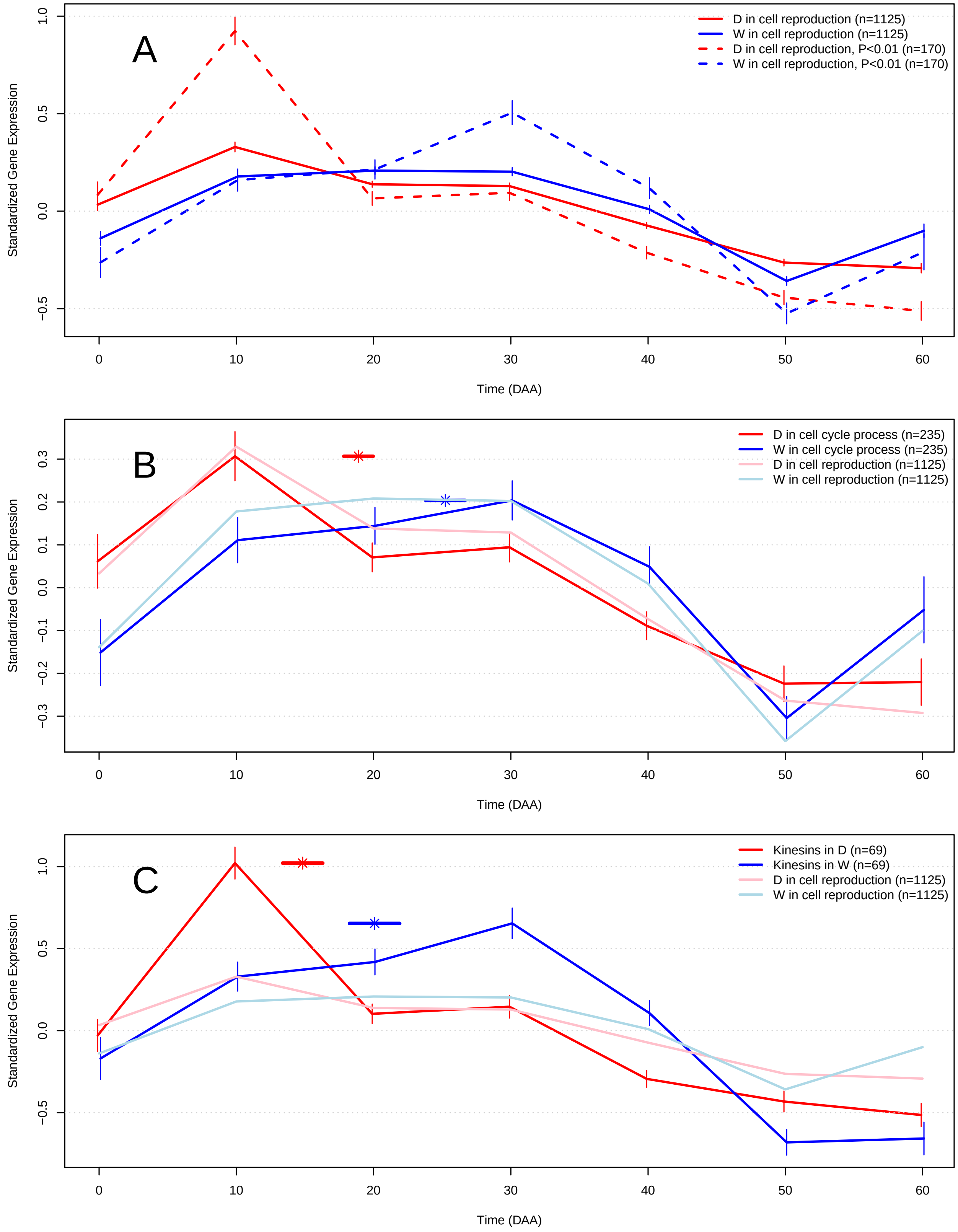

FIGURE 15. **Mean Standardized Expression Profile (SEPs) for groups of genes associated with cell reproduction in Domesticated (D) and Wild (W) accessions.** Vertical lines indicate 95% CI, asterisks denote mean time of maximum expression and horizontal lines over asterisks represent the 95% CI for the parameter. (A) Solid lines show the expression profile for the entire set of 1,125 genes and dashed lines represent expression of a set of 170 genes that had the highest differential expression between the D and W groups ($P < 0.01$). Genes annotated in (B) cell cycle process and (C) Kinesins.

W (Figure 15 B). Similarly, the mean expression for 69 kinesins or kinesin-related proteins among the 1,125 genes associated with cell reproduction exhibited a differential expression peak at 10 DAA for D accessions, but for W accessions the peak was later at 30 DAA (Figure 15 C).

Thus, changes in expression of genes associated with cell reproduction were significantly larger and occurred earlier for D relative to W accessions, not only for the full set of genes, but also for particular bioprocesses and gene families (Figure 15).

S-5.2. **Biological Processes Enriched in Genes That Are Expressed Earlier in Domesticated Genotypes.** The results presented above indicate that SEPs in D and W accessions undoubtedly differ (Figure 14), and genes for which expression peaks at 10 DAA for D but at 30 DAA for W (denoted here as 'D10W30') play an important role in cell reproduction (Figure 15). To validate and expand our study, we considered 542 genes having the D10W30 expression pattern in a Gene Ontology enrichment analysis.

A total of 86 biological processes (BPs) were significantly enriched (FDR = 0.05; $P < 0.0015$) in the D10W30 set, with a median odds ratio of 9.5. As such, these genes were much more abundant in these BPs than would be expected by chance. Apart from the abovementioned BPs related to cell reproduction, 43 of the enriched BPs, or 50% of the total, are involved in either positive or negative regulation of various biological processes. Of these, 4 (5%) are related to cellular component organization or biogenesis, 3 are associated with cellular component assembly, and another 3 play roles in organelle organization or fission. The general bioprocess "cellular process" (GO:0009987) is also highly enriched in the D10W30 gene set, with an odds estimate of 2.25 and a highly significant P-value of 2.76×10^{-8}.

These results show that genes having the pattern D10W30 are over-represented in important BPs, which in turn implies that expression of such BPs occurs earlier and at higher levels in D compared to W genotypes.

These results consider the expression patterns of sets of genes grouped by D and W accessions. Next we considered SEPs for single genes (Figure 16). For the three highlighted genes, the mean expression values for D occur at 10 DAA, while for W the means are observed at 30 DAA, consistent with the pattern D10W30. However, the expression patterns for individual accessions (dotted lines) are variable, even when the mean tendency (continuous lines) consistently followed the D10W30 pattern (Figure 16 A to C).

In examining the expression patterns for the gene encoding the "high mobility group B protein 6", a WRKY transcription factor involved in the nucleosome/chromatin assembly that was annotated in 12 of the 86 abovementioned BPs, particularly cell reproduction BP, there are two outliers among the D10W30 pattern (Figure 16 A). Accession ST (D) had an expression peak at 30 DAA rather than at 10 DAA -even though it had a local maximum at 10 DAA. Accession SY (W) had an expression peak at 40 DAA instead of at 30 DAA. However, the average expression pattern for this gene conforms to the D10W30 pattern and the differences in mean expression between D and W are significant at the two critical points, 10 DAA and 30 DAA.

The gene encoding the transcription factor "MYB-related protein 3R-1" was included in 6 of the 86 enriched BPs and is mainly related to cellular, chromosome and organelle organization. Notably, in comparing Figures 16 A and 16 B, the same accessions, ST (D) and SY (W), are outliers among genes showing the D10W30 pattern, and both had the same tendencies, i.e., high expression at 30 DAA for ST (D) and a late peak at 40 DAA for SY (W). On the other hand, differences in mean expression between D and W were significant at the two critical points 10 DAA and 30 DAA (Figure 16 A, B).

The "kinetochore protein NDC80" is part of multiprotein kinetochore complexes that couple eukaryotic chromosomes to the mitotic spindle to ensure proper chromosome segregation. NDC80 is part of the outer kinetochore and forms a heterotetramer with proteins NUF2, SPC25 and SPC24 (Santaguida and Musacchio, 2009; D?Archivio and Wickstead, 2017). Interestingly, the genes encoding NUF2 and SPC25

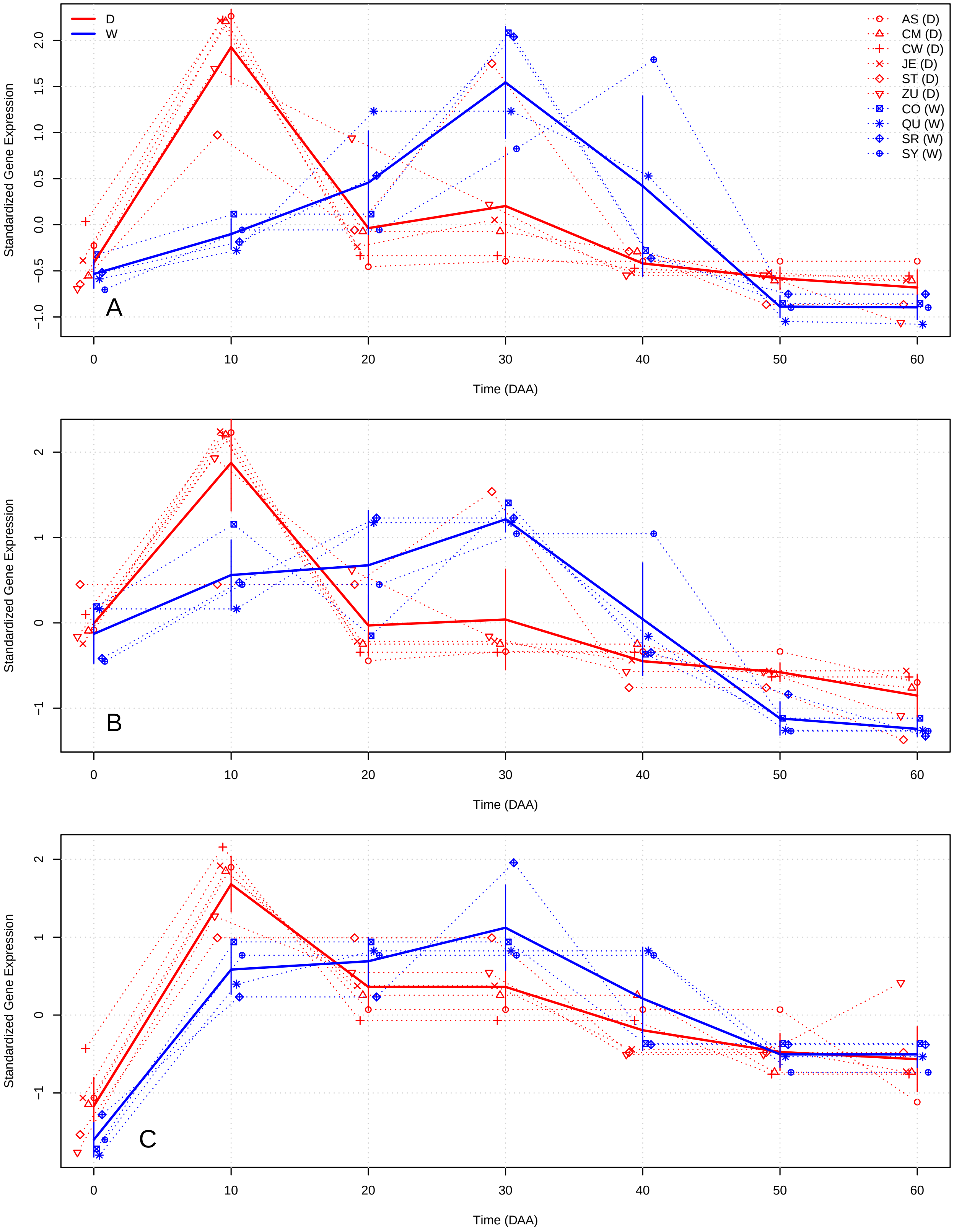

FIGURE 16. **Gene expression patterns for three genes having the D10W30 expression pattern in Domesticated (D) and Wild (W) accessions.** Dashed lines show the SEPs for each accession, and solid lines show mean SEPs per group (D and W). Vertical lines represent 95% CI for mean values at each time. Keys correspond to those shown in Table 1. (A) High mobility group B protein 6 (XP_016555757.1); (B) MYB-related protein 3R-1 (XP_016537977.1); (C) Kinetochore protein NDC80 (XP_016539151.1).

also exhibit the D10W30 expression pattern. NDC80 is conspicuously present in 74 of the 86 enriched BPs (Figure 16 C).

S-6. Gene Ontology (GO) enrichment analyses

After discovering that mean SEP in the D accessions was different to the one in the W group (Figure 1A in the main text), we confronted the problem of finding the functional meaning of that difference, and for this aim we employed Gene Ontology or 'GO' annotations (Ashburner et al., 2000). We isolated the set of genes with a more extreme difference, the $n = 463$ genes with a False Discovery Rate, FDR = 0.05 (Benjamini and Hochberg, 1995), presented in Figure 1B of the main text, and noticed that this group presented the pattern 'D10W30', where the maximum mean expression was at 10 DAA in D, while such maximum occurred at 30 DAA in W (Figure 1B in the main text). Furthermore, we found that a set of 542 genes presented SEPs with D10W30 patterns, and this set was one of the targets for GO enrichment analyses, employing the 'Biological Process' GO ontology and motivated by the results in (Lægreid et al., 2003).

To perform GO enrichment analyses we considered the total population of 22427 genes expressed during fruit development of which 12102 are annotated in one or more of the 2547 GO biological processes annotated in chili. We are interested in the property of a gene to belong to a specific GO category, with the aim to establish whether the class of genes with a specific expression pattern, e.g. genes with mean SEPs D10W30, presented an enrichment in the GO Biological Process of interest with respect to the total gene population. Among the different tests that could be used to test association between a target gene set and a functional GO Biological Process (Rivals et al., 2007), we selected the Fisher's exact test.

We programed a function to summarize the results of the test, and employing different targets performed the analyses of the 2547 GO biological processes, evaluating the P-value of each result, and transforming it to a Q-value to have a FDR (Benjamini and Hochberg, 1995) of 5%. To take into account the structure of the GO ontology, which is fundamental to the analyses interpretation (Rhee et al., 2008), we performed a filtering of redundant and highly correlated biological process using a gene network approach.

As an example of the analyses performed, Appendix S-12.2 presents the R output for the 'Cell Cycle' biological process having as target the set of 542 genes with D10W30 patterns. In Appendix S-12.2 we can see the output of function '`BP.analysis.ById`'. This function gives the observed and expected 2×2 contingency tables as well as the full results of Fisher's exact test, making easier result's interpretation.

Sheet 'Bio Process' in the excel file "`SG.xlsx`" of 'Supplemental Information' presents the full results of the analyses of the 2547 GO biological processes using as target the set of genes with pattern D10W30.

S-7. Genes and Bio Processes (BPs) reported.

Excel file "`SG.xlsx`" in 'Supplemental Information' includes four sheets with the following results:

Gene : Data for the 22427 genes expressed during fruit development (in table "`gene`" of the SALSA database).

Gene column definitions : Column definitions for the "Gene" sheet.

- **id:** Numerical identifier in the SALSA database.
- **ProtId:** Protein identifier of the gene product (if known, otherwise NULL).
- **Prot_Desc:** Protein short description (if known, otherwise NULL).
- **URL:** URL for UniProtKB database using Prot_Desc (if known, otherwise NULL).
- **isTF:** Is the gene product annotated as Transcription Factor? (T if True, F if False).

D10W30: Is the SEP of the gene of class 'D10W30' [see main text] (TRUE or FALSE).

BioProc: Is the gene product annotated in one or more GO Bio Processes (T if True, F if False).

ZunlaDom: Is the gene annotated with domestication footprint in Qin et al. (2014)? NULL if it is not annotated as such, otherwise the name of the gene reported by Qin et al. (2014) is given.

P_value: P value for the test of differences of SEPs between domesticated (D) and wild (W) accessions. See main Methods and Supplemental SI-1.3.

Q_value: P_value transformed to Q_value using R function p.adjust() with method = "fdr" to calculate False Discovery Rate (DFR).

Gene_id: Genomic identifier of the gene.

chromosome: Chromosome where the gene is located; "NULL" if unknown see "scaffold" below.

scaffold: scaffold Scaffold where the gene was located (If Chromosome "NULL").

Strand: Strand coding for the gene ("+" or '-")

start: Genomic coordinate where the gene starts.

end: Genomic coordinate where the gene ends.

length: Length of the gene in base pairs (bps).

sequence: Gene sequence.

Bio Process : Data for the 2547 Gene Ontology (GO) biological processes analyzed (in table "`ResBioProcess`" of the SALSA database).

Bio Process column definitions : Column definitions for the "Bio Process" sheet.

BP.id: Numerical identifier of the Biological Process in the SALSA database.

bio.process: Gene Ontology (GO) Biological Process.

odds: Estimated odds in the contingency table.

P: P-value of the Fisher's exact test for the 2×2 contingency table.

AnnTarg: Number of genes in the process which are annotated in the target.

NotAnnTarg: Number of genes in the process which are NOT annotated in the target.

AnnNotTarg: Number of genes in the process which are annotated but NOT in the target.

NotAnnNotTarg: Number of genes in the process which are NOT annotated and NOT in the target.

Q: P value transformed to Q value using R function p.adjust() with method = "fdr" to calculate False Discovery Rate (DFR).

Information in the "**Gene**" sheet was obtained from the data send by NovoGene after RNA-Seq sequencing and analyses and corresponds to the annotation in the reference genome CM334 v1.6. On the other hand, information in the "**Bio Process**" sheet was the results of the GO enrichment analyses described here in section S-6.

S-8. Network estimation

As mentioned in (Allocco et al., 2004),

> "*It is axiomatic in functional genomics that genes with similar mRNA expression profiles are likely to be regulated via the same mechanisms. This hypothesis is the basis for almost all attempts to use mRNA expression data from microarray experiments to discover regulatory networks.*"

Ideally we would like to estimate a Gene Regulatory Network (GRN) for the whole chili transcriptome. That aim is practically impossible with the current incomplete knowledge of the interactions between genes in the *Capsicum* transcriptome. However, an attainable and relevant goal within the framework of our study is to estimate robust networks of functionally related genes, as the one presented in Figures 3 and 4 of the main text. Here we detail the method employed to obtain that network.

We have a total of 22,427 genes consistently expressed in all accessions, of which 352 are annotated in the BP 'Cell Cycle' and of these 25 belong to the class 'D10W30', i.e., these 25 genes present a maximum expression at 10 DAA in the 6 domesticated (D), while the maximum expression is at 30 DAA in the 4 wild (W) accessions. After examining the Euclidean distances between the SEPs of the 25 genes, we selected 6 of them which present a highly consistent SEPs in both, D and W expression. We selected the 6 structural genes presented in the network of Figure 3 and 4 of the main text (represented by orange circles in that figure) by setting a threshold of Euclidean distance ≤ 1 between pairs of gene SEPs. Table 3 presents the medians of the Pearson correlation ($\hat{r}$) and P values for SEPs of the 6 Structural genes included in the network.

Table 3. Median Pearson Correlation ($\hat{r}$) and P values for SEPs of the 6 Structural genes included in the network presented in Figures 3 and 4 of the main text.

	Between D and W	Within D	Within W
n	144	90	36
$\hat{r}$	0.37477	0.92227	0.77658
P-value	0.40749	0.00310	0.04001
	Between	Within	
n	144	126	
$\hat{r}$	0.37477	0.88496	
P-value	0.40749	0.00810	

In Table 3 column 'Between D and W' presents cases where correlation was estimated for the same gene but taking one D and one W accession, thus correlations are between SEPs in D and W. The number of such pairs of different correlations equals 6 D $\times$ 4 W $\times$ 6 genes, $n = 6 \times 4 \times 6 = 144$. On the other hand, columns 'Within D' and 'Within W' present cases where correlation was estimated for the same gene but taking different accessions within the same group (D or W, respectively). The number of possible comparisons are $n = (6 \times (6-1))/2 \times 6 = 90$ for the column 'Within D' and $n = (4 \times (4-1))/2 \times 6 = 36$ for the column 'Within W'. In Table 3 we can see that the median of the correlations for SEPs within the D and W groups are high, 0.92227 and 0.77658 and significant (P-values of 0.00310 and 0.04001), respectively, while the median of the correlation for SEPs between the D and W groups was smaller, 0.37477, and not significant (P-value of 0.40749). Last rows of Table 3 groups columns 'Within D' and 'Within W' into a single column, 'Within', and from such grouping we obtain the same conclusion than above, i.e., the 6 structural genes have highly and significantly correlated SEPs within but not between accession groups.

Results in Table 3 refer to all posible pairs of the 6 structural genes. However, not all pairs of structural genes are linked (by double headed arrows) in the network of Figures 3 and 4; the genes considered as linked in the network present a value of $\hat{r} > 0.96$ within D and W groups, with a P-value < 0.0001. In

contrast the same pairs of structural genes present a value of $\hat{r} < 0.40$ between D and W groups, with a non-significant P-value > 0.5 Thus the network of structural genes presented in Figures 3 and 4 presents a set of cell cycle genes which are highly coordinated in time within the D and W groups, presenting the expression pattern D10W30.

To corroborate that the expression of the 14 genes included into the network are indeed very well segregated, we performed a Principal Component Analysis (PCA) of the $14 \times 10 = 140$ 7-dimensional SEPs, which tendency by group is presented in Figure 3 B in the main text. In the biplot shown in Figure 17 we see the expression of the 14 genes labeled by their accession of origin and with colors denoting the set of origin (D in red and W in blue), plot in the 2 dimensional space of the first 2 principal components, of which the first (Y axis) explains 57.7% and the second (X axis) explains 17.4% of the data variance, thus together the two first principal components explain 75.1% of the total variance. In this figure we can see that the first principal component efficiently segregates the data into two groups, D in the upper and W in the lower parts of the plot, with only a few outliers. Observing the eigenvectors (dark red arrows in the plot, labeled by the time DAA: 0, 10, $\cdots$, 60) we see that the one at time 0 is almost horizontal, and thus have very small influence in the coordinates transformation. This makes sense, because at 0 DAA both groups (D and W) share the same level of expression, as shown in Figure 3 B in the main text. In contrast all the other 6 eigenvectors, corresponding to times 10, 20, $\cdots$, 60 DAA, have a strong influence in the segregation of the D and W sets, as previously observed in Figure 3 B in the main text.

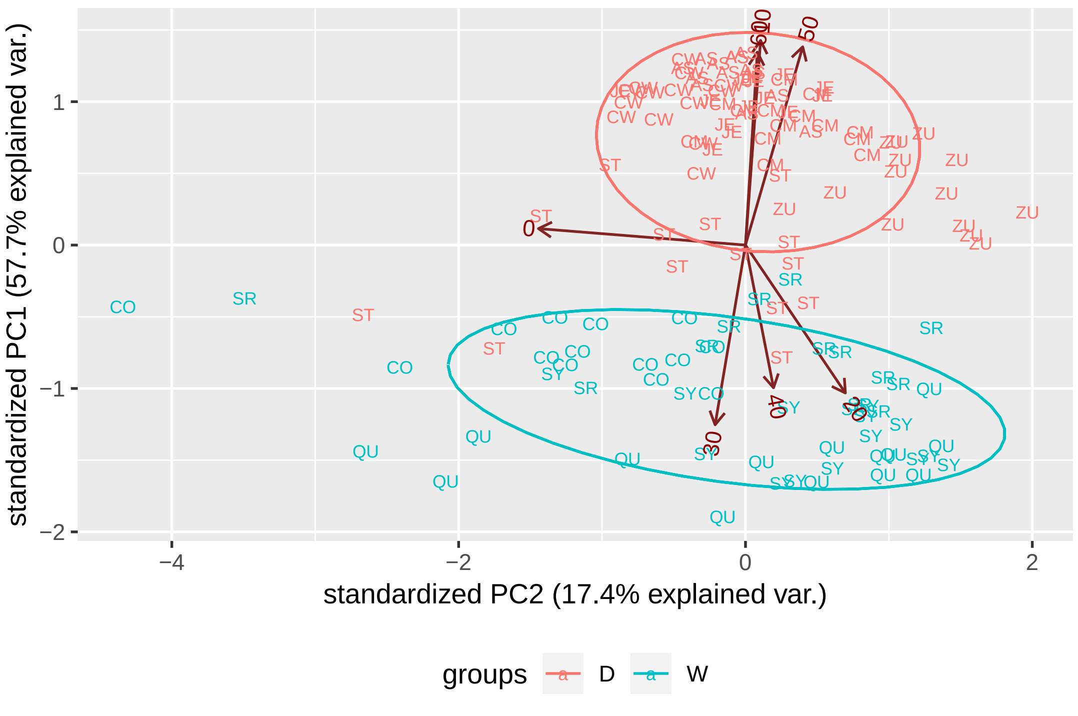

Figure 17. PCA analysis of the SEPs for the 14 genes in the network (Figure 2 B).

S-9. Transcription Factor (TF) imputation

The authors of Allocco et al. (2004) analyzed 611 microarrays and found that the correlation between expression profiles of two genes must be larger than $r \approx 0.84$ to have a 50% chance of sharing a common transcription factor binder. Here we assume that a target gene and a TF which is regulating it will share very alike expression patterns (SEPs) and developed an statistical approach to select a set of candidate TFs. This approach was implemented in an R function which performs the following steps:

Algorithm for TF imputation

(1) Basic input: `id` - Identifier of the target gene; `min.r` - Threshold for the minimum correlation value, $r > 0$; `min.rOm.a` - Threshold for the minimum ratio of r/ma, where ma is the maximum of the absolute difference between the SEPs of the target gene and the SEP of a TF; `acc.set` - The set of accessions where the search will be performed.

(2) Obtains from the database all SEPs for all TFs in all accessions that belong to `acc.set`.

(3) Obtains from the database all SEPs for target gene (`id`) in all accessions that belong to `acc.set`.

(4) For each accession that belong to `acc.set` calculates the correlation, r, and r/ma between the SEPs of each TF and the target gene. Keeps only the cases where $r \geq$ `min.r` AND $r/ma \geq$ `min.rOm.a`.

(5) Obtains a final list of candidate TFs by founding the intersection of all the sets of candidates in each one of the accessions defined int the input (`acc.set`).

(6) Output the list of TFs candidates (if any) as well as variables to judge the adequacy of each TF candidate.

It is important to consider two facts about the above described method. Firstly, parameters $r \geq$ `min.r` AND $r/ma \geq$ `min.rOm.a` are selected in an '*per accession*' base; i.e., they are compared only with the SEPs of the TFs in the same accession. Assume that a given target gene, `id`, is regulated by the same TF, say, `x`, but that target gene has very different expression pattern in two different accessions. If `id` is regulated by `x` in both accessions, the method will likely report `x` in both accessions at step (4), and thus `x` will be part of the final output in (6). Secondly, and more important, given that the data in all accessions are fully independent, the probability of reporting 'erroneous' or 'spurious' TFs decreases exponentially with the number of accessions taken into account. This is, if the probability of reporting a spurious TF in any of the k accessions is ε, then the probability that the procedure reports the same spurious transcription factor in k accessions is ε^k, e.g. if $\varepsilon = 0.5$ and $k = 6$ we have $\varepsilon^k = 0.5^6 \approx 0.016$ and if $k = 10$, $\varepsilon^k = 0.5^{10} \approx 0.001$, etc. Under the null hypothesis of no true correlation between two arbitrary SEPs, the true value of the correlation parameter, ρ, is uniformly distributed in the interval $[-1, 1]$, and if we restrict ourselves to positive values, $\rho \geq 0$, the parameter space is simply $[0, 1]$, and by setting a threshold `min.r` $= 1 - \varepsilon$ and employing k independent accessions in the determination we can effectively fix any desired error probability to be $(1 - \varepsilon)^k$. Furthermore, by additionally asking that $r/ma \geq$ `min.rOm.a` we will filter cases where the correlation, r, is high but at the same time there is an outlier in one of the times, where the maximum of the absolute value, ma, is large. This additional filter adds stringency to the selection method.

After running the algorithm to estimate the TF candidates for each one of the structural genes, we found the 8 TFs which are shown in Figure 3 A as blue circles and in rows 7 to 14 in Table 2 of the main text. The algorithm was run with parameters `min.r = 0.5, min.rOm.a = 0.9` with the full set of 10 accessions. The next box presents the summaries of auxiliar estimates that help to calculate the robustness of the TF candidates.

```
       r                m.a              rOm.a
 Min.   :0.8752   Min.   :0.0924   Min.   : 1.088
 1st Qu.:0.9489   1st Qu.:0.1923   1st Qu.: 1.712
 Median :0.9807   Median :0.3072   Median : 3.211
 Mean   :0.9682   Mean   :0.3747   Mean   : 3.749
 3rd Qu.:0.9931   3rd Qu.:0.5388   3rd Qu.: 5.159
 Max.   :0.9988   Max.   :0.8413   Max.   :10.805
```

The box above summarizes the results for the 8 TFs selected, which are potential regulator of 3 of the structural genes, as shown in Figure 3 A in the main text. The statistics shown are produced from the estimation of $10 \times (4 + 4 + 1) = 90$ cases, that arise because each one of the 3 TFs was evaluated in 10

accessions, and two of them are potential regulators of 4 structural genes and one of them is potential regulator of 1 gene. By taking the mean of the 90 r values, 0.9682, the realized error probability is estimated as $(1-\hat{\varepsilon})^k = (1-0.9682)^{10} = 0.0318^{10} \approx 1.06 \times 10^{-15}$, a vanishing small quantity, thus we can be reasonably sure that the relations found between the structural genes and TFs are, at least for some of the cases, very likely to reflect either, direct or indirect regulation of structural genes by the TF candidates.

The algorithm presented in this section for TF imputation was applied in our data to nominate TF candidates for the AT3 gene, resulting in the selection of only two TF, precisely the ones that have been experimentally validated as regulators of AT3 (Arce-Rodríguez and Ochoa-Alejo, 2017; Zhu et al., 2019; Sun et al., 2019). The fact that our approach recovers experimentally validated TFs demonstrates that this approach retrieves strong TFs candidates.

S-10. Supplementary descriptions and web links for genes in the network

Descriptions

Items in this list give a short description and references for genes in the network of Figures 3 A and 4 and Table 2 in the main text. In each case the *Capsicum* protein identifier from Table 4 is followed by the putative *Arabidopsis* ortholog between parenthesis. Order in this list is the same than the one presented in Table 2 of the main text as well as in the rows of tables 4 and 5 presented below.

(1) XP_016564755.1 (AT5G51600) 65-kDa microtubule-associated protein 3 (MAP65/ASE1). Members of the AtMAP65 family –to which AT5G51600 belongs, link membrane and microtubule dynamics during plant cytokinesis, the part of the cell division process during which the cytoplasm of a single cell divides into two daughter cells. It appears that these proteins are required to coordinate cytokinesis with the nuclear division cycle, and some MAP65 family members are known to be targets of cell cycle-regulated kinases (Steiner et al., 2016).

(2) XP_016538322.1 (AT2G44190) QWRF motif-containing protein 6 (DUF566). It has been demonstrated that ENDOSPERM DEFECTIVE1 (EDE1), a mutant of the AT2G44190 gene, is expressed in the endosperm and embryo of developing seeds, and its expression is tightly regulated during cell cycle progression (Pignocchi et al., 2009). Furthermore, the authors show that EDE1 protein accumulates in nuclear caps in premitotic cells, colocalizes along microtubules of the spindle and phragmoplast, and binds microtubules in vitro. The aforementioned paper concludes that this gene codes for a microtubule-associated protein (DUF566), essential for seed development in *Arabidopsis*.

(3) XP_016541615.1 (AT4G21270) Kinesin 3 isoform X3 (kinesin 1). The spindle is critical for chromosome segregation, and kinesins play crucial roles in spindle structure; in particular the *Arabidopsis* ATK1 gene (AT4G21270) is required for spindle morphogenesis in male meiosis (Chen et al., 2002). Even when XP_016541615.1 is identified as kinesin 3 (row 3 in Table 4), it is more alike with the kinesin 1 of *Arabidopsis* (alignments obtained by blastx in Appendix S-12.1) and thus it is identified with AT4G21270 in Table 5.

(4) XP_016575449.1 (AT5G51600); see item (1) in this list and (Steiner et al., 2016).

(5) XP_016577799.1 (AT4G20900) Protein POLLENLESS 3 (TPR). Members of the tetratricopeptide repeat (TPR) superfamily had been found in cell cycle clusters during apple fruit development (Janssen et al., 2008) and it had been demonstrated that their expression is highly regulated in early developing that fruit (Soria-Guerra et al., 2011).

(6) XP_016548908.1 (AT3G44960) Shugoshin. Shugoshin protects the sister chromatid cohesion complex (cohesin) for proper chromosome segregation in mitosis, until kinetochores are properly captured by the spindle microtubules (Kitajima et al., 2006)

(7) XP_016568750.1 (AT5G42700) B3 domain protein (AP2/B3-like transcriptional factor family protein) The plant-specific B3 superfamily includes families, such as the auxin response factor (ARF) family and the LAV family, as well as less well understood families, such as RAV and REM. There are indications that the B3 domain evolved on the plant lineage before multicellularity (Swaminathan et al., 2008), and, for example, the over-expression of an *Arabidopsis* B3 TF, ABS2/NGAL1 leads to the loss of flower petals (Shao et al., 2012).

(8) XP_016555757.1 (AT4G11080) High mobility group B protein 6 (HMG). The high mobility group B protein 6, belongs to the HMG (high mobility group) box proteins, which is a group of chromosomal proteins that are involved in the regulation of DNA-dependent processes such as transcription, replication, recombination, and DNA repair (Johns, 2012).

(9) XP_016543946.1 (AT3G11520) G2/mitotic-specific cyclin S13-7 (CYCLIN B1;3) is a regulatory protein involved in mitosis and, importantly, it is first activated in the cytoplasm and that centrosomes may function as sites of integration for the proteins that trigger mitosis (Jackman et al., 2003).

(10) XP_016547461.1 (AT1G26760) B3 domain-containing protein (SET domain protein 35). AT1G26760 received high scores for plastids localization (Schwacke et al., 2007), and has been also reported in maintaining H3K4 methylation (Liu and Gong, 2011).

(11) XP_016575946.1 (AT5G58280) B3 domain-containing protein At5g58280; AP2/B3-like transcriptional factor family protein. This gene has been reported to be differentially expressed in the flower and seed in *Brassica rapa*, castor bean, cocoa, soybean, and maize (Peng and Weselake, 2013), with tissues of preferential expression of the orthologous B3 gene pairs in Arabidopsis and rice.

(12) XP_016574880.1 (AT1G34355) FHA domain-containing protein PS1; forkhead-associated (FHA) domain-containing protein. An insertional mutation of AT1G34355, the AtPS1 gene has been characterized and found to lead to the production of diploid pollen grains (d'Erfurth et al., 2008).

(13) XP_016537977.1 (AT4G32730) Myb-related protein 3R-1 (Homeodomain-like protein). In plants, this class of Myb proteins are believed to regulate the transcription of G2/M phase-specific genes; in particular MYB3R1 act as transcriptional activator and positively regulate cytokinesis. In addition, MYB3R1 may play an important role during fruit development by regulating G2/M-specific genes (Haga et al., 2011).

(14) XP_016565918.1 (AT3G22780). Protein tesmin/TSO1 CXC 3; Tesmin/TSO1-like CXC domain-containing protein. TSO1 is a protein that modulates cytokinesis and cell expansion in *Arabidopsis* (Hauser et al., 2000).

Table 4. NCBI links and descriptions of genes in Figure 3 A and Table 2 in the main text.

Row	id	Prot. Id (link)	Short Protein Description
1	673	XP_016564755.1	65-kDa microtubule-associated protein 3
2	6090	XP_016538322.1	QWRF motif-containing protein 6
3	15446	XP_016541615.1	kinesin 3 isoform X3
4	19658	XP_016575449.1	65-kDa microtubule-associated protein 3 isoform X1
5	19813	XP_016577799.1	protein POLLENLESS 3
6	24546	XP_016548908.1	shugoshin-1; meiotic chromosome segregation
7	19147	XP_016568750.1	B3 domain Prot. At5g42700
8	24186	XP_016555757.1	high mobility group B protein 6
9	35149	XP_016543946.1	G2/mitotic-specific cyclin S13-7
10	5824	XP_016547461.1	SET domain; methyltransferase activity; LOC107847605
11	11410	XP_016575946.1	B3 domain-containing protein At5g58280
12	12656	XP_016574880.1	FHA domain-containing protein PS1
13	13605	XP_016537977.1	Myb-related protein 3R-1
14	7175	XP_016565918.1	protein tesmin/TSO1 CXC 3

Table 5. Putative *Arabidopsis* orthologous of genes in Figure 3 A and Table 2 in the main text.

Row	id	NCBI id	TAIR id	Short Protein Description.
1	673	NP_199973.1	AT5G51600	Microtubule associated protein (MAP65/ASE1).
2	6090	NP_181947.1	AT2G44190	ENDOSPERM DEFECTIVE protein (DUF566).
3	15446	NP_193859.1	AT4G21270	Kinesin 1
4	19658	NP_199973.1	AT5G51600	Microtubule associated protein (MAP65/ASE1).
5	19813	NP_001328331.1	AT4G20900	Tetratricopeptide repeat (TPR)-like superfamily.
6	24546	NP_001319686.1	AT3G44960	Shugoshin
7	19147	NP_001318733.1	AT5G42700	AP2/B3-like transcriptional factor family protein
8	24186	NP_192846.1	AT4G11080	HMG (high mobility group) box protein
9	35149	NP_187759.2	AT3G11520	CYCLIN B1;3
10	5824	NP_173998.2	AT1G26760	SET domain protein 35
11	11410	NP_001330080.1	AT5G58280	AP2/B3-like transcriptional factor family protein
12	12656	NP_001320842.1	AT1G34355	forkhead-associated (FHA) domain-containing protein
13	13605	NP_001328944.1	AT4G32730	Homeodomain-like protein
14	7175	NP_566718.2	AT3G22780	Tesmin/TSO1-like CXC domain-containing protein

References

Abdi H (2007) Bonferroni and šidák corrections for multiple comparisons. *Encyclopedia of measurement and statistics*, 3, 103–107.

Allocco DJ, Kohane IS, and Butte AJ (2004) Quantifying the relationship between co-expression, co-regulation and gene function. *BMC bioinformatics*, 5, 1–10.

Arce-Rodríguez ML and Ochoa-Alejo N (2017) An r2r3-myb transcription factor regulates capsaicinoid biosynthesis. *Plant physiology*, 174, 1359–1370.

Ashburner M, Ball CA, Blake JA, Botstein D, Butler H, Cherry JM, Davis AP, Dolinski K, Dwight SS, Eppig JT, et al. (2000) Gene ontology: tool for the unification of biology. *Nature genetics*, 25, 25–29.

Benjamini Y and Hochberg Y (1995) Controlling the false discovery rate: a practical and powerful approach to multiple testing. *Journal of the royal statistical society. Series B (Methodological)*, pp. 289–300.

Bland M (2013) Do baseline P-values follow a uniform distribution in randomised trials? *PloS one*, 8, e76010.

Chen C, Marcus A, Li W, Hu Y, Calzada JPV, Grossniklaus U, Cyr RJ, and Ma H (2002) The arabidopsis atk1 gene is required for spindle morphogenesis in male meiosis. *Development*, 129, 2401–2409.

D'Archivio S and Wickstead B (2017) Trypanosome outer kinetochore proteins suggest conservation of chromosome segregation machinery across eukaryotes. *Journal of Cell Biology*, 216, 379–391.

d'Erfurth I, Jolivet S, Froger N, Catrice O, Novatchkova M, Simon M, Jenczewski E, and Mercier R (2008) Mutations in atps1 (arabidopsis thaliana parallel spindle 1) lead to the production of diploid pollen grains. *PLoS Genet*, 4, e1000274.

Edgar R, Domrachev M, and Lash AE (2002) Gene expression omnibus: Ncbi gene expression and hybridization array data repository. *Nucleic acids research*, 30, 207–210.

Escoto-Sandoval C, Flores-Díaz A, Reyes-Valdés MH, Ochoa-Alejo N, and Martinez O (2020) An R package for data mining chili pepper fruit transcriptomes. *(in evaluation)*. doi:10.21203/rs.3.rs-130806/v1. URL `https://www.researchsquare.com/article/rs-130806/v1`.

Gómez-García MdR and Ochoa-Alejo N (2013) Biochemistry and molecular biology of carotenoid biosynthesis in chili peppers (*Capsicum* spp.). *International journal of molecular sciences*, 14, 19025–19053.

Haga N, Kobayashi K, Suzuki T, Maeo K, Kubo M, Ohtani M, Mitsuda N, Demura T, Nakamura K, Jürgens G, et al. (2011) Mutations in myb3r1 and myb3r4 cause pleiotropic developmental defects and preferential down-regulation of multiple g2/m-specific genes in arabidopsis. *Plant Physiology*, 157, 706–717.

Hauser BA, He JQ, Park SO, and Gasser CS (2000) Tso1 is a novel protein that modulates cytokinesis and cell expansion in arabidopsis. *Development*, 127, 2219–2226.

Iglesias-Martinez LF, Kolch W, and Santra T (2016) Bgrmi: A method for inferring gene regulatory networks from time-course gene expression data and its application in breast cancer research. *Scientific Reports*, 6.

Jackman M, Lindon C, Nigg EA, and Pines J (2003) Active cyclin b1–cdk1 first appears on centrosomes in prophase. *Nature cell biology*, 5, 143–148.

Janssen BJ, Thodey K, Schaffer RJ, Alba R, Balakrishnan L, Bishop R, Bowen JH, Crowhurst RN, Gleave AP, Ledger S, et al. (2008) Global gene expression analysis of apple fruit development from the floral bud to ripe fruit. *BMC Plant Biology*, 8, 16.

Johns E (2012) *The Chromosomal Proteins*. Elsevier.

Kitajima TS, Sakuno T, Ishiguro Ki, Iemura Si, Natsume T, Kawashima SA, and Watanabe Y (2006) Shugoshin collaborates with protein phosphatase 2a to protect cohesin. *Nature*, 441, 46–52.

Kothari S, Joshi A, Kachhwaha S, and Ochoa-Alejo N (2010) Chilli peppers?a review on tissue culture and transgenesis. *Biotechnology advances*, 28, 35–48.

Lægreid A, Hvidsten TR, Midelfart H, Komorowski J, and Sandvik AK (2003) Predicting gene ontology biological process from temporal gene expression patterns. *Genome research*, 13, 965–979.

Liu Q and Gong Z (2011) The coupling of epigenome replication with dna replication. *Current opinion in plant biology*, 14, 187–194.

Luan Y and Li H (2003) Clustering of time-course gene expression data using a mixed-effects model with b-splines. *Bioinformatics*, 19, 474–482.

Martínez O, Arce-Rodríguez ML, Hernández-Godínez F, Escoto-Sandoval C, Cervantes-Hernández F, Hayano-Kanashiro C, Ordaz-Ortiz JJ, Reyes-Valdés MH, Razo-Mendivil FG, Garcés-Claver A, and Ochoa-Alejo N (2020) Transcriptomic analyses throughout chili pepper fruit development reveal novel insights into domestication process. *bioRxiv*. doi:10.1101/2020.10.05.326470. URL `https://www.biorxiv.org/content/early/2020/10/09/2020.10.05.326470`.

Martínez O and Escoto-Sandoval C (2021) *Salsa: An R package of data mining facilities for Capsicum gene expression profiles*. doi:10.5281/zenodo.4587745. URL `https://doi.org/10.5281/zenodo.4587745`. This research was funded by the Consejo Nacional de Ciencia y Tecnología, México (Conacyt) project number 1570.

Martínez-López LA, Ochoa-Alejo N, and Martínez O (2014) Dynamics of the chili pepper transcriptome during fruit development. *BMC genomics*, 15, 143.

Mortazavi A, Williams BA, McCue K, Schaeffer L, and Wold B (2008) Mapping and quantifying mammalian transcriptomes by rna-seq. *Nature methods*, 5, 621.

Peng FY and Weselake RJ (2013) Genome-wide identification and analysis of the b3 superfamily of transcription factors in brassicaceae and major crop plants. *Theoretical and Applied Genetics*, 126, 1305–1319.

Peng RD (2011) Reproducible research in computational science. *Science*, 334, 1226–1227.

Pignocchi C, Minns GE, Nesi N, Koumproglou R, Kitsios G, Benning C, Lloyd CW, Doonan JH, and Hills MJ (2009) Endosperm defective1 is a novel microtubule-associated protein essential for seed development in arabidopsis. *The Plant Cell*, 21, 90–105.

Qin C, Yu C, Shen Y, Fang X, Chen L, Min J, Cheng J, Zhao S, Xu M, Luo Y, et al. (2014) Whole-genome sequencing of cultivated and wild peppers provides insights into capsicum domestication and specialization. *Proceedings of the National Academy of Sciences*, 111, 5135–5140.

R Core Team (2013) *R: A language and environment for statistical computing*. R Foundation for Statistical Computing, Vienna, Austria. URL `http://www.r-project.org`.

Rhee SY, Wood V, Dolinski K, and Draghici S (2008) Use and misuse of the gene ontology annotations. *Nature Reviews Genetics*, 9, 509.

Rivals I, Personnaz L, Taing L, and Potier MC (2007) Enrichment or depletion of a go category within a class of genes: which test? *Bioinformatics*, 23, 401–407.

Robinson MD, McCarthy DJ, and Smyth GK (2010) edgeR: a bioconductor package for differential expression analysis of digital gene expression data. *Bioinformatics*, 26, 139–140.

Santaguida S and Musacchio A (2009) The life and miracles of kinetochores. *The EMBO journal*, 28, 2511–2531.

Schwacke R, Fischer K, Ketelsen B, Krupinska K, and Krause K (2007) Comparative survey of plastid and mitochondrial targeting properties of transcription factors in arabidopsis and rice. *Molecular Genetics and Genomics*, 277, 631–646.

Shao J, Liu X, Wang R, Zhang G, and Yu F (2012) The over-expression of an arabidopsis b3 transcription factor, abs2/ngal1, leads to the loss of flower petals. *PloS one*, 7, e49861.

Singh DK and McNellis TW (2011) Fibrillin protein function: the tip of the iceberg? *Trends in plant science*, 16, 432–441.

Soria-Guerra RE, Rosales-Mendoza S, Gasic K, Wisniewski ME, Band M, and Korban SS (2011) Gene expression is highly regulated in early developing fruit of apple. *Plant Molecular Biology Reporter*, 29, 885.

Steiner A, Rybak K, Altmann M, McFarlane HE, Klaeger S, Nguyen N, Facher E, Ivakov A, Wanner G, Kuster B, et al. (2016) Cell cycle-regulated pleiade/at map 65-3 links membrane and microtubule dynamics during plant cytokinesis. *The Plant Journal*, 88, 531–541.

Sun B, Zhu Z, Chen C, Chen G, Cao B, Chen C, and Lei J (2019) Jasmonate-inducible r2r3-myb transcription factor regulates capsaicinoid biosynthesis and stamen development in capsicum. *Journal of agricultural and food chemistry*, 67, 10891–10903.

Swaminathan K, Peterson K, and Jack T (2008) The plant b3 superfamily. *Trends in plant science*, 13, 647–655.

Tian SL, Li L, Shah S, and Gong ZH (2015) The relationship between red fruit colour formation and key genes of capsanthin biosynthesis pathway in capsicum annuum. *Biologia plantarum*, 59, 507–513.

Wang Z, Gerstein M, and Snyder M (2009) RNA-Seq: a revolutionary tool for transcriptomics. *Nature reviews genetics*, 10, 57–63.

Zhu Z, Sun B, Cai W, Zhou X, Mao Y, Chen C, Wei J, Cao B, Chen C, Chen G, et al. (2019) Natural variations in the myb transcription factor myb31 determine the evolution of extremely pungent peppers. *New Phytologist*, 223, 922–938.

S-11. Appendix (R output)

S-12. Analyses of gene with id=580 (FBN); see Figure 11 which presents the plot obtained with the function.

```
----------------------------------------------------------------------------------------
> TMmean.plot(580)

 Means of 10 TMs in 6 D and 4 W accessions
(1 different genes)

Function call:  TMmean.plot 580

alpha = 0.05 All Confidence Intervals (CI) at 95%.

Means and CI for Standardized expression per time in D

      ne.0 ne.10 ne.20 ne.30 ne.40 ne.50 ne.60
Mean -0.53 -0.62 -0.53 -0.52 -0.37  0.76  1.81
LL   -0.62 -0.71 -0.65 -0.60 -0.49  0.08  1.52
UL   -0.44 -0.54 -0.41 -0.44 -0.25  1.44  2.11
```

```
Means and CI for Standardized expression per time in W

      ne.0 ne.10 ne.20 ne.30 ne.40 ne.50 ne.60
Mean -0.63 -0.62 -0.59 -0.57 -0.40  1.01  1.79
LL   -0.70 -0.68 -0.62 -0.64 -0.41  0.64  1.54
UL   -0.56 -0.55 -0.56 -0.49 -0.38  1.37  2.05

P-values for the t-test of means D vs W per time point:
  ne.0  ne.10  ne.20  ne.30  ne.40  ne.50  ne.60
0.1196 0.9104 0.4017 0.4331 0.6729 0.5471 0.9198

Summary of those P-values:
   Min. 1st Qu.  Median    Mean 3rd Qu.    Max.
 0.1196  0.4174  0.5471  0.5721  0.7917  0.9198

Estimation of the point in time (DAA)
of maximum Standardized expression in D
  LCL  mean   UCL
52.53 56.67 60.80

Estimation of the point in time (DAA)
of maximum Standardized expression in W
 LCL mean  UCL
52.6 57.5 62.4

Estimated difference between maxima in D and W: -0.83 DAA

(Genes are Early in D  but the difference is NOT significant at 0.05)

T-test for the difference of maxima expression between D and W

Welch Two Sample t-test

data:  D.max.perTM and W.max.perTM
t = -0.25482, df = 6.739, p-value = 0.8065
alternative hypothesis: true difference in means is not equal to 0
95 percent confidence interval:
 -8.627334  6.960667
sample estimates:
mean of x mean of y
 56.66667  57.50000
---------------------------------------------------------------------------------------
```

S-12.1. **Analyses of gene with id= 19147 (B3 domain-containing protein); see Figure 13 which presents the plot obtained with the function.**

```
---------------------------------------------------------------------------------------
> TMmean.plot(19147)

 Means of 10 TMs in 6 D and 4 W accessions
(1 different genes)
```

```
Function call:  TMmean.plot 19147

alpha = 0.05 All Confidence Intervals (CI) at 95%.

Means and CI for Standardized expression per time in D

      ne.0 ne.10 ne.20 ne.30 ne.40 ne.50 ne.60
Mean -0.35  2.14 -0.24 -0.06 -0.46 -0.48 -0.54
LL   -0.52  1.95 -0.48 -0.56 -0.54 -0.55 -0.63
UL   -0.19  2.32  0.00  0.45 -0.38 -0.41 -0.45

Means and CI for Standardized expression per time in W

      ne.0 ne.10 ne.20 ne.30 ne.40 ne.50 ne.60
Mean -0.43  0.25   0.3  1.73  0.08 -0.96 -0.97
LL   -0.71 -0.15  -0.4  1.38 -0.27 -1.09 -1.11
UL   -0.14  0.65   1.0  2.08  0.43 -0.83 -0.84

P-values for the t-test of means D vs W per time point:
  ne.0  ne.10  ne.20  ne.30  ne.40  ne.50  ne.60
0.6861 0.0008 0.2311 0.0005 0.0535 0.0016 0.0026

Summary of those P-values:
     Min.   1st Qu.    Median      Mean   3rd Qu.      Max.
0.0004559 0.0012052 0.0025650 0.1394492 0.1423184 0.6860762

Estimation of the point in time (DAA)
of maximum Standardized expression in D
 LCL mean  UCL
  10   10   10

Estimation of the point in time (DAA)
of maximum Standardized expression in W
 LCL mean  UCL
22.6 27.5 32.4

Estimated difference between maxima in D and W: -17.5 DAA

(Genes are Early in D )

Note: maxima in D and W are uniform
(thus no t-test was possible)
----------------------------------------------------------------------------------
```

S-12.2. **Analyses of GO biological process "Cell Cycle" having as target the D10W30 set of genes.**

```
----------------------------------------------------------------------------------
# Running function 'BP.analysis.ById' and printing results
> BP.analysis.ById(D10W30.ids, BP.id=207)
Number of ids in target:  542
```

```
In accessions:  All
Biological Process: cell cycle

Observed matrix:
        Target NotTarget
Annot       25       327
NoAnnot    282     11444

Rounded expected values:
        Target NotTarget
Annot     8.95    343.05
NoAnnot 298.05  11427.95

Estimated odds ratio from the table:
 3.102566

Fisher's Exact Test for Count Data

data:  temp.t
p-value = 3.513e-06
alternative hypothesis: true odds ratio is not equal to 1
95 percent confidence interval:
 1.944868 4.758091
sample estimates:
odds ratio
  3.102053

# Running function 'BP.analysis.ById' without printing results
# (for further analysis of groups of biological processes)
> temp <- BP.analysis.ById(D10W30.ids, BP.id=207, print.all=FALSE)
> temp
  Acc BP.id bio.process     odds            P AnnTarg NotAnnTarg AnnNotTarg
1 All   207  cell cycle 3.102566 3.512919e-06      25        282        327
  NotAnnNotTarg
1         11444
-------------------------------------------------------------------------------------
```

S-13. Appendix (Clean reads per library and genotype)

Table 6. Number of clean reads map to the reference genome (n, in millions).

Library	Accession	Type	Time	Replicate	n
AS00R1	AS	D	00	R1	13.52
AS00R2	AS	D	00	R2	15.36
AS10R1	AS	D	10	R1	14.60
AS10R2	AS	D	10	R2	14.95
AS20R1	AS	D	20	R1	16.03
AS20R2	AS	D	20	R2	17.10
AS30R1	AS	D	30	R1	13.30
AS30R2	AS	D	30	R2	17.62
AS40R1	AS	D	40	R1	15.79
AS40R2	AS	D	40	R2	17.46
AS50R1	AS	D	50	R1	15.91
AS50R2	AS	D	50	R2	18.39
AS60R1	AS	D	60	R1	15.42
AS60R2	AS	D	60	R2	18.20
CM00R1	CM	D	00	R1	21.05
CM00R2	CM	D	00	R2	20.59
CM10R1	CM	D	10	R1	15.27
CM10R2	CM	D	10	R2	19.20
CM20R1	CM	D	20	R1	17.17
CM20R2	CM	D	20	R2	17.90
CM30R1	CM	D	30	R1	16.98
CM30R2	CM	D	30	R2	14.83
CM40R1	CM	D	40	R1	15.36
CM40R2	CM	D	40	R2	15.51
CM50R1	CM	D	50	R1	23.86
CM50R2	CM	D	50	R2	18.41
CM60R1	CM	D	60	R1	15.16
CM60R2	CM	D	60	R2	18.39
CO00R1	CO	W	00	R1	16.36
CO00R2	CO	W	00	R2	16.91
CO10R1	CO	W	10	R1	13.66
CO10R2	CO	W	10	R2	14.12
CO20R1	CO	W	20	R1	13.21
CO20R2	CO	W	20	R2	17.44
CO30R1	CO	W	30	R1	16.97
CO30R2	CO	W	30	R2	15.88
CO40R1	CO	W	40	R1	17.83
CO40R2	CO	W	40	R2	18.73
CO50R1	CO	W	50	R1	19.79
CO50R2	CO	W	50	R2	18.19
CO60R1	CO	W	60	R1	18.05
CO60R2	CO	W	60	R2	15.04
CW00R1	CW	D	00	R1	18.75
CW00R2	CW	D	00	R2	20.00
CW10R1	CW	D	10	R1	19.90
CW10R2	CW	D	10	R2	15.64

Table 7. Continue. Number of clean reads map to the reference genome (n, in millions).

Library	Accession	Type	Time	Replicate	n
CW20R1	CW	D	20	R1	23.69
CW20R2	CW	D	20	R2	12.60
CW30R1	CW	D	30	R1	19.60
CW30R2	CW	D	30	R2	17.14
CW40R1	CW	D	40	R1	20.87
CW40R2	CW	D	40	R2	18.85
CW50R1	CW	D	50	R1	18.72
CW50R2	CW	D	50	R2	19.75
CW60R1	CW	D	60	R1	19.26
CW60R2	CW	D	60	R2	18.42
JE00R1	JE	D	00	R1	16.76
JE00R2	JE	D	00	R2	18.94
JE10R1	JE	D	10	R1	17.68
JE10R2	JE	D	10	R2	15.18
JE20R1	JE	D	20	R1	16.69
JE20R2	JE	D	20	R2	17.14
JE30R1	JE	D	30	R1	16.36
JE30R2	JE	D	30	R2	15.85
JE40R1	JE	D	40	R1	15.16
JE40R2	JE	D	40	R2	17.01
JE50R1	JE	D	50	R1	15.18
JE50R2	JE	D	50	R2	17.00
JE60R1	JE	D	60	R1	18.86
JE60R2	JE	D	60	R2	15.86
QU00R1	QU	W	00	R1	14.95
QU00R2	QU	W	00	R2	18.86
QU10R1	QU	W	10	R1	15.68
QU10R2	QU	W	10	R2	16.81
QU20R1	QU	W	20	R1	16.09
QU20R2	QU	W	20	R2	15.85
QU30R1	QU	W	30	R1	16.17
QU30R2	QU	W	30	R2	17.10
QU40R1	QU	W	40	R1	16.18
QU40R2	QU	W	40	R2	14.27
QU50R1	QU	W	50	R1	12.72
QU50R2	QU	W	50	R2	14.78
QU60R1	QU	W	60	R1	15.48
QU60R2	QU	W	60	R2	15.57
SR00R1	SR	W	00	R1	16.26
SR00R2	SR	W	00	R2	15.03
SR10R1	SR	W	10	R1	13.54
SR10R2	SR	W	10	R2	18.27
SR20R1	SR	W	20	R1	19.19
SR20R2	SR	W	20	R2	15.53
SR30R1	SR	W	30	R1	17.70
SR30R2	SR	W	30	R2	18.09
SR40R1	SR	W	40	R1	18.13
SR40R2	SR	W	40	R2	14.66
SR50R1	SR	W	50	R1	14.07
SR50R2	SR	W	50	R2	16.71

Table 8. Continue. Number of clean reads map to the reference genome (n, in millions).

Library	Accession	Type	Time	Replicate	n
SR60R1	SR	W	60	R1	12.71
SR60R2	SR	W	60	R2	14.03
ST00R1	ST	D	00	R1	12.10
ST00R2	ST	D	00	R2	13.69
ST10R1	ST	D	10	R1	13.96
ST10R2	ST	D	10	R2	12.12
ST20R1	ST	D	20	R1	13.85
ST20R2	ST	D	20	R2	15.03
ST30R1	ST	D	30	R1	14.80
ST30R2	ST	D	30	R2	14.38
ST40R1	ST	D	40	R1	13.81
ST40R2	ST	D	40	R2	17.47
ST50R1	ST	D	50	R1	16.17
ST50R2	ST	D	50	R2	18.30
ST60R1	ST	D	60	R1	14.24
ST60R2	ST	D	60	R2	15.28
SY00R1	SY	W	00	R1	15.06
SY00R2	SY	W	00	R2	13.68
SY10R1	SY	W	10	R1	17.19
SY10R2	SY	W	10	R2	18.11
SY20R1	SY	W	20	R1	19.74
SY20R2	SY	W	20	R2	20.16
SY30R1	SY	W	30	R1	18.87
SY30R2	SY	W	30	R2	16.99
SY40R1	SY	W	40	R1	12.75
SY40R2	SY	W	40	R2	13.90
SY50R1	SY	W	50	R1	10.33
SY50R2	SY	W	50	R2	13.37
SY60R1	SY	W	60	R1	14.17
SY60R2	SY	W	60	R2	16.82
ZU00R1	ZU	D	00	R1	15.35
ZU00R2	ZU	D	00	R2	13.99
ZU10R1	ZU	D	10	R1	16.49
ZU10R2	ZU	D	10	R2	13.94
ZU20R1	ZU	D	20	R1	16.89
ZU20R2	ZU	D	20	R2	11.59
ZU30R1	ZU	D	30	R1	18.23
ZU30R2	ZU	D	30	R2	14.24
ZU40R1	ZU	D	40	R1	22.64
ZU40R2	ZU	D	40	R2	14.61
ZU50R1	ZU	D	50	R1	17.83
ZU50R2	ZU	D	50	R2	16.77
ZU60R1	ZU	D	60	R1	16.25
ZU60R2	ZU	D	60	R2	16.85

Table 9. Number of clean reads map to the reference genome per genotype (n, in millions).

		Statistics for n (in millions).					
Genotype	Libraries	Total	min	median	mean	max	S
AS	14	223.63	13.30	15.85	15.97	18.39	1.61
CM	14	249.67	14.83	17.54	17.83	23.86	2.66
CO	14	232.17	13.21	16.94	16.58	19.79	1.98
CW	14	263.18	12.60	19.05	18.80	23.69	2.54
JE	14	233.67	15.16	16.72	16.69	18.94	1.23
QU	14	220.50	12.72	15.77	15.75	18.86	1.42
SR	14	223.92	12.71	15.89	15.99	19.19	2.06
ST	14	205.20	12.10	14.31	14.66	18.30	1.76
SY	14	221.14	10.33	15.94	15.80	20.16	2.91
ZU	14	225.68	11.59	16.37	16.12	22.64	2.60
D	84	1401.04	11.59	16.59	16.68	23.86	2.47
W	56	897.74	10.33	16.13	16.03	20.16	2.13
Total	140	2298.78	10.33	16.26	16.42	23.86	2.35
